# EFTUD2 Regulates Cortical Morphogenesis via Modulation of *Caspase‐3* and *Aifm1* Splicing Pathways

**DOI:** 10.1002/advs.202504200

**Published:** 2025-05-31

**Authors:** Liping Chen, Ying Li, Yan Yu, Mingze Cai, Hao Li, Minghe Huang, Guochao Yang, Jiageng Guo, Huailin Wang, Zhihong Song, Wei Shen, Huihui Jiang, Haitao Wu

**Affiliations:** ^1^ Department of Neurobiology Beijing Institute of Basic Medical Sciences Beijing 100850 China; ^2^ Key Laboratory of Neuroregeneration Co‐innovation Center of Neuroregeneration Nantong University Nantong Jiangsu Province 226019 China; ^3^ Chinese Institute for Brain Research Beijing 102206 China

**Keywords:** alternative splicing, Aifm1, cortical morphogenesis, EFTUD2, mandibulofacial dysostosis‐microcephaly syndrome

## Abstract

Elongation Factor Tu GTP‐Binding Domain Containing 2 (EFTUD2), a core spliceosomal GTPase associated with Mandibulofacial Dysostosis with Microcephaly (MFDM), plays a mechanistically undefined role in cerebral development. To investigate its pathophysiological contributions, murine models are generated through conditional *Eftud2* ablation and in utero electroporation of human pathogenic *EFTUD2* variants into cortical neural stem cells (NSCs). Embryonic NSC‐specific *Eftud2* knockout resulted in cortical disorganization and microcephaly, while pathogenic variants led to significant neuronal loss. Integrative transcriptomic and immunofluorescence analyses revealed that *Eftud2* deficiency triggers apoptotic pathways, contributing to cortical malformations. Mechanistic studies using RNA co‐immunoprecipitation and full‐length transcriptome sequencing demonstrated that Eftud2 directly interacts with *Caspase3* and *Aifm1* transcripts, regulating their alternative splicing to generate pro‐apoptotic isoforms. Splicing assays functionally validated this regulatory mechanism, showing its role in activating cell death pathways and disrupting neurodevelopmental homeostasis. These findings elucidate EFTUD2's critical role in maintaining apoptotic balance during corticogenesis and identify defective splicing regulation as the molecular basis of MFDM. This study provides insights for advancing diagnostic frameworks and therapeutic strategies for neurodevelopmental disorders.

## Graphical abstract

EFTUD2, a spliceosomal GTPase linked to MFDM, regulates cortical development through apoptotic control. Conditional *Eftud2* knockout in murine neural stem cells induces microcephaly and cortical disorganization, while pathogenic variants drive neuronal loss. Multi‐omics analyses show Eftud2 deficiency activates apoptosis by directly binding *Caspase3*/*Aifm1* pre‐mRNAs, promoting pro‐apoptotic isoform splicing. This defective RNA processing underlies neurodevelopmental imbalances, elucidating MFDM pathogenesis and guiding therapeutic strategies for splicing‐related disorders.

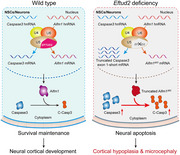

## 1 Introduction

Cortical morphogenesis is a highly coordinated spatiotemporal process involving neuronal proliferation, differentiation, migration, and apoptotic regulation‐each critical for the establishment of functional neural circuitry.^[^ ^1^, ^2^ ^]^ Dysregulation of these processes underlies the pathogenesis of various neurodevelopmental disorders characterized by cortical malformations^[^ ^3^, ^4^ ^]^ Mandibulofacial Dysostosis with Microcephaly (MFDM), an autosomal dominant disorder presenting with microcephaly, craniofacial dysmorphism, and neurological deficits,^[^ ^5^, ^6^ ^]^ is primarily caused by heterozygous mutations in the spliceosomal GTPase *EFTUD2*.^[^ ^7^, ^8^ ^]^ While 70% of MFDM cases result from *de novo EFTUD2* mutations,^[^ ^9^ ^]^ the molecular mechanisms linking these genetic alterations to phenotypic manifestations remain poorly understood, impeding the development of targeted therapies.

Alternative splicing, a key mechanism generating transcriptomic and proteomic diversity in eukaryotes,^[^ ^10^ ^]^ plays a critical role in neurodevelopment. Splicing defects have been implicated in microcephaly, epileptogenesis, and cognitive impairment.^[^ ^11^ ^]^ This process is regulated by spliceosomes, complex molecular machines essential for precise splicing.^[^ ^12^ ^]^ EFTUD2 (Elongation Factor Tu GTP‐Binding Domain Containing 2/Snu114), a GTPase component of the spliceosome,^[^ ^8^, ^13^, ^14^ ^]^ not only orchestrates splicing but also regulates immunity, oncogenesis, and tissue differentiation.^[^ ^15^, ^16^, ^17^, ^18^, ^19^, ^20^, ^21^ ^]^ Perturbation studies in model organisms highlight its conserved neurodevelopmental functions: zebrafish *eftud2* mutants exhibit encephalic malformations,^[^ ^22^ ^]^ while murine neural crest‐specific or Purkinje‐specific deletions recapitulate MFDM craniofacial phenotypes and induce cerebellar hypoplasia, respectively.^[^ ^16^, ^23^, ^24^ ^]^ Notably, elevated *Eftud2* expression in murine progenitor populations corresponding to human MFDM‐affected regions suggests dosage‐sensitive roles in cortical development,^[^ ^23^ ^]^ though the underlying mechanisms remain incompletely understood.

Current models propose both Trp53‐dependent and Trp53‐independent mechanisms in EFTUD2‐related pathology. Zebrafish *eftud2* mutants and murine neural crest knockouts demonstrate Trp53‐mediated apoptosis driving craniofacial hypoplasia.^[^ ^16^, ^22^ ^]^ However, targeted knockout of exon 2 in the murine *Eftud2* gene induces MFDM‐like phenotypes in homozygous mice without activating the Trp53 pathway.^[^ ^23^ ^]^ Furthermore, craniofacial defects in embryos with homozygous *Eftud2* deletion in neural crest cells are not rescued by *Trp53* deletion.^[^ ^25^ ^]^ Similarly, *Trp53* deletion fails to prevent Purkinje cell death and cerebellar abnormalities caused by *Eftud2* deficiency.^[^ ^24^ ^]^ Intriguingly, Eftud2 has been shown to inhibit ferroptosis, thereby maintaining Purkinje cell survival through a Trp53‐independent mechanism.^[^ ^24^ ^]^ These findings underscore the complexity of EFTUD2's roles in brain development and highlight its diverse molecular targets. Further investigation is essential to elucidate the precise mechanisms by which EFTUD2 regulates these processes and contributes to neurodevelopmental disorders such as MFDM.

This study employs a combination of genetic and molecular approaches to define the role of *Eftud2* in murine cortical development. Using conditional knockout models and pathogenic mutant expression in neural stem cells (NSCs), we demonstrate that *Eftud2* deficiency disrupts cortical histogenesis through NSC and neuronal depletion. Transcriptomic and histopathological analyses reveal apoptotic activation as the primary pathogenic mechanism. Mechanistic studies integrating isoform sequencing (Iso‐Seq) and RNA‐protein interaction mapping establish that *Eftud2* directly regulates the alternative splicing of apoptotic effectors *Caspase3* and *Aifm1* (Apoptosis‐inducing factor mitochondria‐associated 1). Our findings highlight a splicing‐dependent pathway through which EFTUD2 maintains cortical integrity, providing a molecular framework for understanding MFDM pathogenesis.

## 2 Results

### 2.1 Pathogenic EFTUD2 Variants Impair Functionality and Induce Cortical Neuronal Loss

≈70% of MFDM cases result from *de novo EFTUD2* mutations, as documented in the *EFTUD2* Mutation Database http://databases.lovd.nl/shared/genes/EFTUD2).^[^ ^26^ ^]^ A meta‐analysis of clinically reported MFDM‐associated mutations (Figure S1A, Supporting Information) revealed strong evolutionary conservation across mammalian species, including *Homo sapiens*, *Pan troglodytes*, and *Mus musculus* (Figure S1B, Supporting Information), indicating that these variants localize to critical functional domains.

To investigate the neurodevelopmental consequences of human *EFTUD2* mutations, we selected four clinically documented missense variants with cross‐species conservation (Figure S1B, Supporting Information). Structural modeling indicated that R262, L637, and A823 sites are located in solvent‐exposed regions, while C476 occupies a buried core position (Figure S2A–D, Supporting Information), providing a structural basis for potential functional impairment. Despite these alterations, mutant proteins retained wild‐type stability and nuclear localization in cortical neurons (Figure S2E–G, Supporting Information), suggesting that their pathogenicity arises from functional disruptions rather than global structural instability or mislocalization.

**Figure 1:**
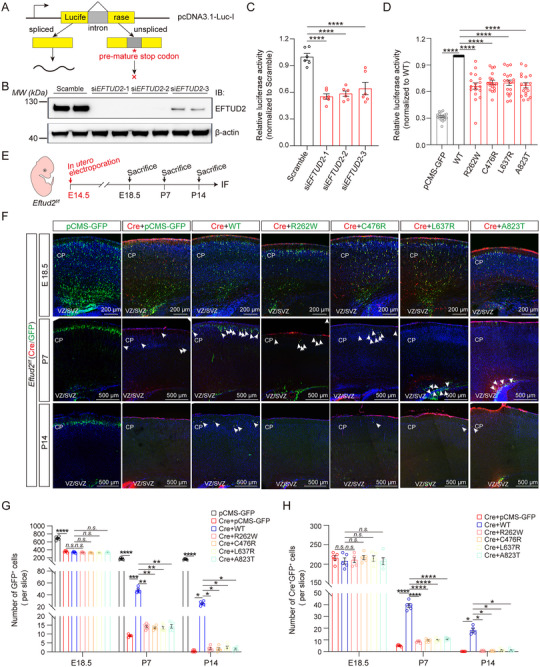
Pathogenic EFTUD2 mutations impair spliceosomal activity and induce cortical neuronal loss. A) Schematic diagram of the luciferase‐intron reporter construct (pcDNA3.1‐Luc‐I) used to assess RNA splicing efficiency. B) Immunoblot validation of *EFTUD2* knockdown in HEK293T cells transfected with scramble siRNA or three independent siRNAs targeting *EFTUD2* (si*EFTUD2*‐1, si*EFTUD2*‐2, si*EFTUD2*‐3). β‐actin served as the loading control. C) Luciferase activity in HEK293T cells treated with siRNA for 48 h and co‐transfected with pcDNA3.1‐Luc‐I or pcDNA3.1‐Luc and the Renilla luciferase reporter pRL‐TK for normalization. Activity was normalized to the Scramble group (*n* = 6; one‐way ANOVA and Dunnett's multiple comparisons test). D) Relative luciferase activity in HEK293T cells co‐transfected for 48 h with the pCMS‐GFP vector, wild‐type *EFTUD2* (WT) or mutant *EFTUD2* constructs (R262W, C476R, L637R, A823T) alongside pcDNA3.1‐Luc‐I and pRL‐TK. Results show a significant reduction in activity for all mutants compared to WT (*n* = 18; one‐way ANOVA and Tukey multiple comparisons test). E) Experimental timeline for in utero electroporation and phenotypic analysis. F) Representative confocal images of the cortical ventricular/subventricular zone (VZ/SVZ) and cortical plate (CP) showing mCherry‐Cre^+^ (knockout), GFP^+^ (rescue), and double‐positive cells. Scale bars: 200 µm, 500 µm. G) Quantification of GFP‐positive cells in electroporated cortical sections within the VZ/SVZ and CP (*n* = 5 mice; two‐way ANOVA and Tukey multiple comparisons test). H) Quantification of double‐positive cells in electroporated cortical sections within the VZ/SVZ and CP (*n* = 5 mice; two‐way ANOVA and Tukey multiple comparisons test). All data represent means ± SEM. *p < 0.05, **p < 0.01, ***p < 0.001, ****p < 0.0001, n.s., not significant. Abbreviations: CP, cortical plate; VZ, ventricular zone; SVZ, subventricular zone.

To assess the effects of pathogenic *EFTUD2* mutations on RNA splicing fidelity, we developed a fluorescent reporter plasmid (pcDNA3.1‐Luc‐I) containing a 132‐nucleotide intronic element from β‐immunoglobulin, where proper splicing is required for luciferase activation.^[^ ^27^, ^28^ ^]^ This system establishes a positive correlation between fluorescence intensity and splicing efficiency, as impaired intron excision prevents luciferase transcript maturation (**Figure** **1**A). Initial validation using siRNA‐mediated *EFTUD2* knockdown in HEK293T cells confirmed effective protein depletion (Figure 1B) and a significant reduction in luciferase activity (Figure 1C). Further functional studies also supported this relationship: co‐transfection of wild‐type (WT) *EFTUD2* with the reporter construct restored basal splicing activity, whereas all pathogenic mutants (R262W, C476R, L637R, A823T) exhibited significantly impaired rescue capacity (Figure 1D). Quantitative analysis revealed 30–40% reduction in relative luciferase units for mutants compared to WT controls (Figure 1D), confirming their loss‐of‐function nature in RNA processing.

Given EFTUD2's essential GTPase activity in spliceosomal function,^[^ ^29^, ^30^ ^]^ we characterized the enzymatic activity of mutant proteins. Biochemical assays showed substantial impairment in GTP hydrolysis for R262W, C476R, and L637R mutants compared to WT, while A823T retained 97% of WT activity (Figure S3A, Supporting Information). This differential impairment aligns with structural localization data, as R262 resides within the catalytic GTP‐binding domain (Figure S2A, Supporting Information). EFTUD2 interacts with U5 RNA and spliceosomal proteins, including PRPF8, PRPF6, and SNRNP200, to form the U5 spliceosome complex.^[^ ^13^, ^14^ ^]^ PRPF8, a major structural protein, directly binds EFTUD2.^[^ ^31^, ^32^ ^]^ Co‐immunoprecipitation assays revealed significant binding deficits between PRPF8 and C476R or L637R mutants, whereas R262W and A823T maintained near‐WT interaction capacity (Figure S3B–E, Supporting Information). These findings suggest distinct mechanistic pathways: R262W mutant primarily disrupts catalytic function through GTPase impairment, while C476R and L637R mutants compromise structural integration with core spliceosomal components.

To determine the neurodevelopmental consequences of *EFTUD2* dysfunction, we utilized a conditional knockout murine model (*Eftud2^f/f^*) combined with in utero electroporation to study cortical neuron survival dynamics (Figure 1E). The introduction of Cre ablates endogenous Eftud2 in cortical neurons of *Eftud2^f/f^* mice, resulting in a significant reduction of GFP‐positive cells compared to the pCMS‐GFP‐only control group, indicating that endogenous Eftud2 knockout in mice leads to the loss of cortical neurons (Figure 1F,G). Concurrently, this approach enabled Cre recombinase‐mediated ablation of endogenous *Eftud2* alongside ectopic expression of human WT *EFTUD2* and pathogenic variants (R262W, C476R, L637R, A823T). Quantitative analysis revealed progressive neurodegeneration, with Eftud2 ablation reducing neuronal survival at P7 and P14 (Figure 1F–H). While WT human *EFTUD2* ectopic expression rescued neuronal populations, all pathogenic *EFTUD2* mutants failed to prevent neurodegeneration (Figure 1F–H). These in vivo findings demonstrate the complete loss‐of‐function nature of the mutants and provide direct evidence linking *EFTUD2* splicing defects to cortical hypoplasia in MFDM.

### 2.2 NSC‐Specific *Eftud2* Ablation Recapitulates MFDM Neuropathology Through Neural Stem Cell and Neuronal Depletion

Central nervous system (CNS) developmental anomalies are hallmark features of MFDM,^[^ ^33^ ^]^ with congenital microcephaly indicating prenatal origin of cerebral malformations.^[^ ^6^, ^9^, ^34^ ^]^ Pathogenic missense mutations in *EFTUD2* have been identified as loss‐of‐function variants (Figure 1), and together with clinically reported nonsense and frameshift mutations, these findings strongly suggest that EFTUD2 contributes to MFDM through a loss‐of‐function mechanism. During various stages of embryonic development (E13.5–E18.5) in mice, Eftud2 is ubiquitously expressed in the cerebral cortex, with particularly elevated expression in neural stem cells (NSCs) located in the ventricular and subventricular zones (VZ/SVZ), as well as in neurons of the developing cortical plate (Figure S4A, Supporting Information). To elucidate the molecular pathogenesis underlying microcephaly, we generated NSC‐restricted *Eftud2* knockout murine models using hGFAP‐Cre‐mediated recombination, achieving targeted gene ablation from embryonic day 12.5 (E12.5).^[^ ^35^, ^36^ ^]^ Comparative immunofluorescence and immunoblot analyses of neonatal cortices confirmed substantial *Eftud2* depletion in *hGFAP‐Cre; Eftud2^f/f^* (*Eftud2^hGFAP^*) mice compared to controls (*Eftud2^f/f^*) (Figure S4B–D, Supporting Information), validating the successful establishment of NSC‐specific knockout lineages.

**Figure 2:**
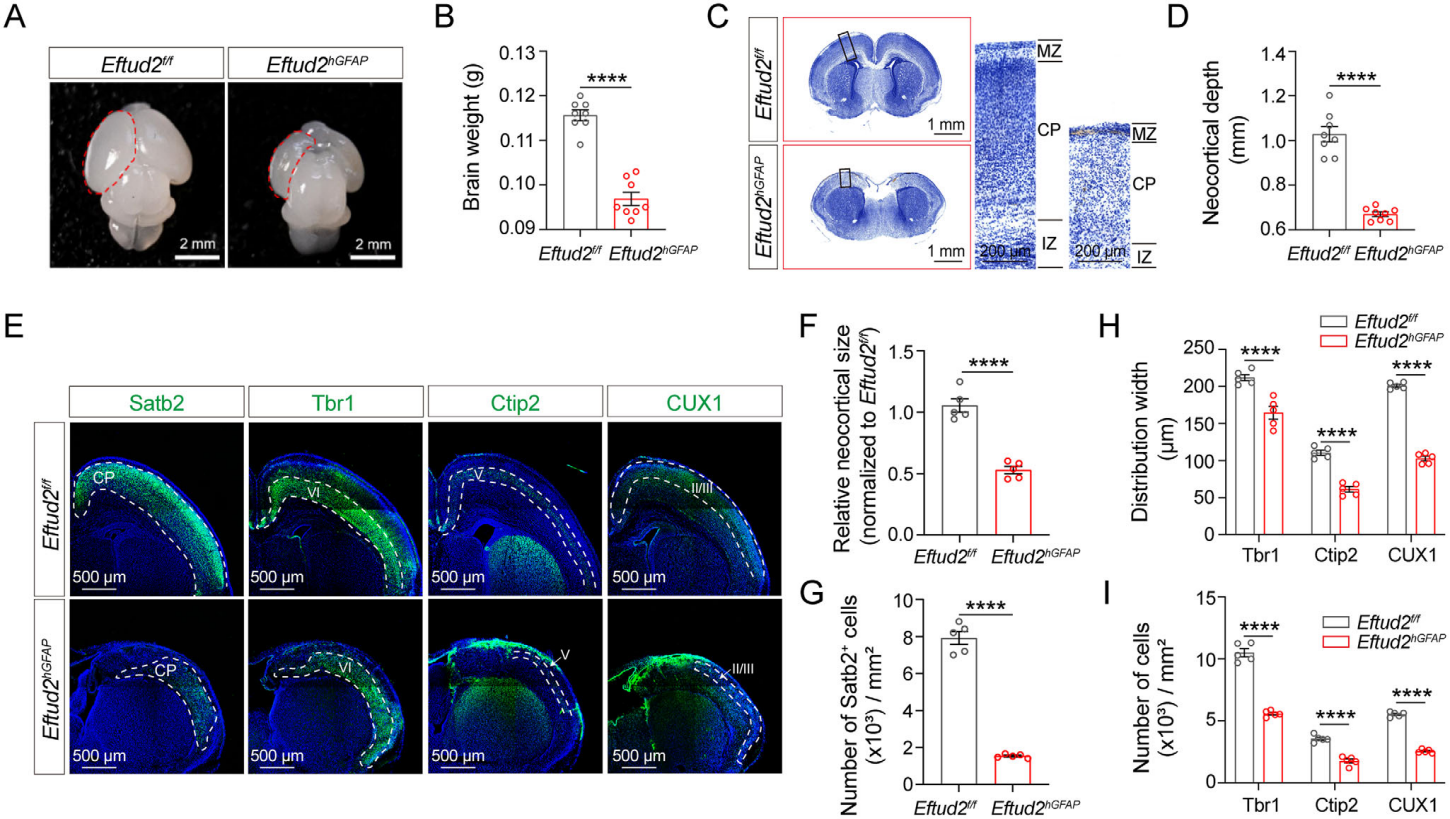
Neural stem cell‐specific *Eftud2* ablation induces cortical hypoplasia. A) Macroscopic view of postnatal day 0 (P0) brains from *Eftud2^f/f^* (control) and *Eftud2^hGFAP^* (cKO) mice. Scale bar: 2 mm. B) Quantification of whole‐brain weight reveals a significant reduction in cKO mice compared to controls (*n* = 8 mice; unpaired *t*‐test). C) Nissl‐stained coronal sections showing cortical thinning in cKO mice (boxed regions). Scale bars: 1 mm (overview), 200 µm (insets). D) Cortical thickness measurement highlights thinner cortices in cKO mice (boxed areas) (*n* = 8 mice; unpaired *t*‐test). Scale bars: 1 mm (whole section) and 200 µm (magnified region). E) Immunofluorescence staining shows decreased expression of the neocortical marker Satb2, deep‐layer markers Tbr1 (layer VI) and Ctip2 (layer V), and superficial‐layer marker CUX1 (layers II/III) in the cortices of cKO mice at P0. Scale bar: 500 µm. F) Quantification of relative neocortical size demonstrates a significant reduction in cKO mice (*n* = 5 mice; unpaired *t*‐test). G) Quantification of Satb2+ cells per unit area in the neocortex shows significant loss in cKO mice (*n* = 5 mice; unpaired *t*‐test). H) Distribution width of neocortical markers per unit area is significantly reduced in cKO mice (*n* = 5 mice; two‐way ANOVA and Sidak multiple comparisons test). I) Total cell density in the neocortex per unit area is significantly reduced in cKO mice (*n* = 5 mice; two‐way ANOVA and Sidak multiple comparisons test). All data represent means ± SEM. ****p < 0.0001. Abbreviations: MZ, marginal zone; CP, cortical plate; IZ, intermediate zone.

Neonatal *Eftud2^hGFAP^* mice exhibited significant reductions in encephalic mass compared to littermate controls (*Eftud2^f/f^*) (**Figure** **2**A–B). Histomorphometric analysis using Nissl staining revealed profound neural architectural disturbances in *Eftud2^hGFAP^* mice, including severe cortical deformities, a substantial reduction in cortical area, and an ≈40% decrease in cortical depth (Figure 2C,D). Immunostaining for Satb2, a marker of the neocortex, demonstrated reduced cortical regions and neuronal density in *Eftud2^hGFAP^* mice compared to controls (Figure 2E–G). Stratification analysis using layer‐specific biomarkers (CUX1 for layers II‐III; Ctip2 for layer V; and Tbr1 for layer VI) revealed disrupted laminar organization, characterized by diminished distribution breadth and cellular density across all cortical layers in *Eftud2^hGFAP^* mice (Figure 2E,H,I). These findings collectively demonstrate that NSC‐specific *Eftud2* ablation recapitulates key neuropathological features of MFDM through impaired corticogenesis.

To further investigate *Eftud2*’s role in cortical neurogenesis, we generated *Emx1‐Cre; Eftud2^f/f^* conditional knockout mice (*Eftud2^Emx1^*). *Emx1‐Cre*‐mediated recombination targets dorsal pallial progenitors beginning at embryonic day 10.5 (E10.5), coinciding with the onset of early cortical progenitor proliferation.^[^ ^37^ ^]^ Nissl staining of brain sections revealed a significant reduction in cortical thickness in *Eftud2^Emx1^* mice compared to control littermates (Figure S5A,B, Supporting Information). Similarly, the Satb2^+^ cortical regions in *Eftud2^Emx1^* mice were reduced to ≈70% of those observed in control mice (Figure S5C,D, Supporting Information). Laminar disorganization extended to Ctip2^+^ layer V and Tbr1^+^ layer VI neurons, which exhibited aberrant dispersion patterns in *Eftud2^Emx1^* mice (Figure S5C,D, Supporting Information), further corroborating *Eftud2*'s conserved role across corticogenic models.

Given that *Eftud2* depletion in early cortical NSCs leads to severe disruptions in cortical size and architecture, we next investigated the underlying mechanisms driving cortical developmental defects in *Eftud2^hGFAP^* mice during embryonic stages. Nissl staining revealed significant reductions in cortical area and size in *Eftud2^hGFAP^* mice starting at embryonic day 15.5 (E15.5) (Figure S6A–C, Supporting Information). The period between E13.5 and E15.5 is critical for NSC proliferation and differentiation. Immunostaining for Sox2, a marker of NSCs, showed a significant reduction in NSC numbers in *Eftud2^hGFAP^* mice by E15.5 (Figure S6D,E, Supporting Information). Correspondingly, the number of mature cortical neurons derived from NSCs was also markedly reduced in *Eftud2^hGFAP^* mice compared to controls (Figure S6F,G, Supporting Information). Concurrent with these findings, quantitative analysis revealed a marked reduction in EGFR+ glial progenitor cell populations within the cerebral cortices of *Eftud2^hGFAP^* cKO mice relative to controls (Figure S7, Supporting Information). This observed depletion of EGFR‐expressing progenitors indicates that Eftud2 deficiency disrupts gliogenic potential in the *Eftud2^hGFAP^* mice. These findings suggest that the cortical developmental abnormalities and microcephaly‐like phenotype observed in *Eftud2*‐deficient mice are primarily driven by a significant reduction in NSCs, neuronal and glial cell populations. This conclusion aligns with the neuronal loss phenotype resulting from defective *EFTUD2* function due to clinical missense mutations, as described earlier.

### 2.3 Eftud2 Depletion Triggers Apoptotic Cascades in Cortical Neural Populations

**Figure 3:**
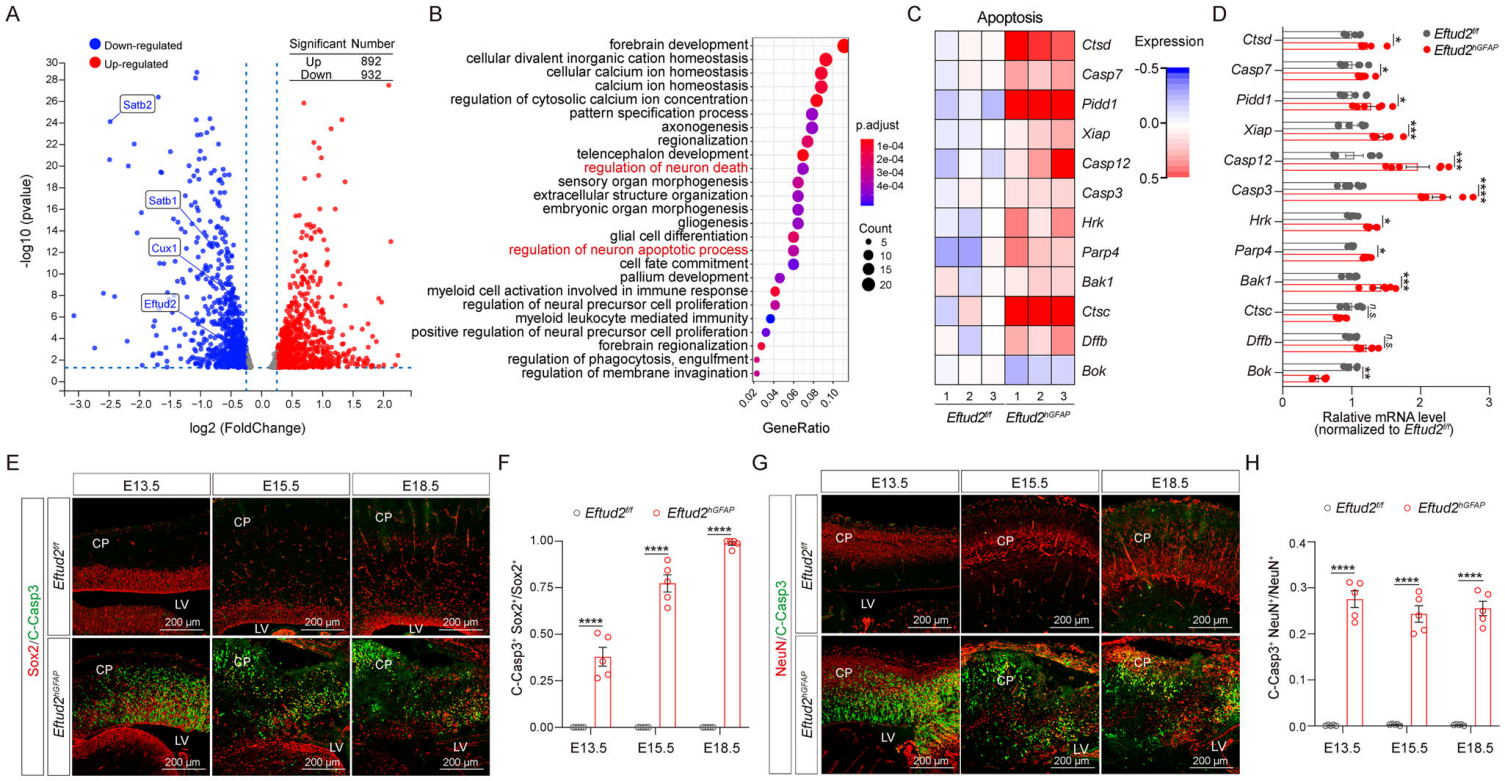
*Eftud2* deficiency activates apoptotic pathways in cortical neural populations. A) Volcano plot of differentially expressed genes in the cortex of *Eftud2^hGFAP^* (cKO) mice. B) Gene Ontology (GO) enrichment analysis highlights apoptosis‐related pathways in the cortex of cKO mice. C) Heatmap of apoptosis signaling pathway gene expression, standardized by z‐scores (*n* = 3; unpaired *t*‐test). The top bar indicates sample genotypes. D) Quantitative RT‐PCR validation reveals significant changes in the expression of apoptosis‐related genes in the cortex of *Eftud2^f/f^* and *Eftud2^hGFAP^* mice (*n* = 6; unpaired *t*‐test). E) Representative immunofluorescent images showing NSC marker Sox2 and apoptosis marker cleaved‐Caspase3 (C‐Casp3) in the cortex of *Eftud2^f/f^* and *Eftud2^hGFAP^* mice at E13.5, E15.5, and E18.5. Scale bar: 200 µm. F) Quantification of the C‐Casp3^+^ NSCs in the cortex of *Eftud2^f/f^* and *Eftud2^hGFAP^* mice at E13.5, E15.5, and E18.5 (*n* = 5 mice; two‐way ANOVA and Sidak multiple comparisons test). G) Representative immunofluorescent images showing neuron marker NeuN and apoptosis marker C‐Casp3 in the cortex of *Eftud2^f/f^* and *Eftud2^hGFAP^* mice at E13.5, E15.5, and E18.5. Scale bar: 200 µm. H) Quantification of apoptotic neuron ratios in the cortex of *Eftud2^f/f^* and *Eftud2^hGFAP^* mice at E13.5, E15.5, and E18.5 (*n* = 5 mice; two‐way ANOVA and Sidak multiple comparisons test). All data represent means ± SEM. *p < 0.05, **p < 0.01, ***p < 0.001, ****p < 0.0001, n.s., not significant. Abbreviations: CP, cortical plate; LV, lateral ventricle.

To elucidate the molecular mechanisms by which *Eftud2* regulates NSCs and cortical development, we performed transcriptomic profiling via RNA sequencing (RNA‐seq) of neonatal cortices (P0). The analysis revealed substantial transcriptional dysregulation following *Eftud2* ablation, with 1824 differentially expressed genes (892 upregulated; 932 downregulated; FDR ≤ 5%, |log2FC| ≥ 0.25) in *Eftud2^hGFAP^* mice compared to *Eftud2^f/f^* controls (**Figure** **3**A). Gene Ontology (GO) enrichment analysis highlighted significant perturbations in developmental processes, particularly forebrain morphogenesis and calcium homeostasis, consistent with the neurogenesis deficiencies observed in *Eftud2^hGFAP^* mice (Figure 2; Figures S5 and S6, Supporting Information). Notably, pathways associated with neuron death and apoptotic signaling were prominently affected, with marked upregulation of terminal apoptotic mediators such as *Ctsd*, *Casp7*, *Pidd1*, *Casp12*, and *Caspase3* (Figure 3B,C). Quantitative PCR validation confirmed elevated transcriptional activation of apoptosis‐associated genes (Figure 3D), suggesting that *Eftud2* plays a regulatory role in programmed cell death pathways during corticogenesis.

To further substantiate these findings, we conducted immunohistochemical analysis of apoptotic markers across developmental stages. Cortical sections from *Eftud2^f/f^* and *Eftud2^hGFAP^* mice were examined for cleaved‐Caspase3 (C‐Casp3), Sox2, and NeuN. Quantitative analysis revealed a pronounced increase in C‐Casp3 immunoreactivity in both Sox2^+^ NSCs and NeuN^+^ neurons throughout the E13.5‐E18.5 developmental window (Figure 3E–H). Terminal deoxynucleotidyl transferase (TdT) dUTP Nick‐End Labeling (TUNEL) staining further confirmed increased apoptosis in the *Eftud2^hGFAP^* cortex, as evidenced by a significant rise in TUNEL^+^ apoptotic cells (Figure S8A,B, Supporting Information). Additionally, immunoblot quantification demonstrated markedly elevated levels of C‐Casp3 protein in the cortex of *Eftud2^hGFAP^* mice (Figure S8C,D, Supporting Information). Similar observations in *Eftud2^Emx1^* models revealed analogous Caspase3 activation patterns, with cortical apoptotic indices elevated 15‐fold compared to wild‐type littermates (Figure S8E,F, Supporting Information). Collectively, these data indicate that Eftud2 prevents apoptosis in NSCs during early development, thereby sustaining cortical neurogenesis.

To investigate the neurotoxic effects of pathogenic *EFTUD2* variants, we employed embryonic electroporation to achieve concurrent endogenous *Eftud2* ablation and ectopic expression of mutant *EFTUD2* in cortical neural populations. Spatial mapping of cleaved‐Caspase3 expression revealed mutation‐specific apoptotic activation. Pathogenic *EFTUD2* missense mutations significantly upregulated cleaved‐Caspase3 levels in both NSCs and mature neurons (Figure S9, Supporting Information). Notably, the R262W and C476R mutations primarily induced apoptosis in NSCs, whereas the L637R and A823T mutations predominantly triggered apoptosis in mature neurons within the neocortex (Figure S9, Supporting Information). This cellular compartment‐specific vulnerability suggests mutation‐dependent mechanisms that warrant further mechanistic investigation.

In summary, *Eftud2* serves as a critical regulator of apoptotic homeostasis in developing neural populations. Loss‐of‐function mutations disrupt neurodevelopmental homeostasis through cell type‐selective Caspase activation, leading to the depletion of both progenitor cells and differentiated neurons. This pathophysiological cascade underlies cortical hypoplasia in MFDM and related microcephalic disorders.

### 2.4 *Eftud2* Regulates Apoptotic Pathways via Alternative Splicing of *Caspase3* and *Aifm1*

**Figure 4:**
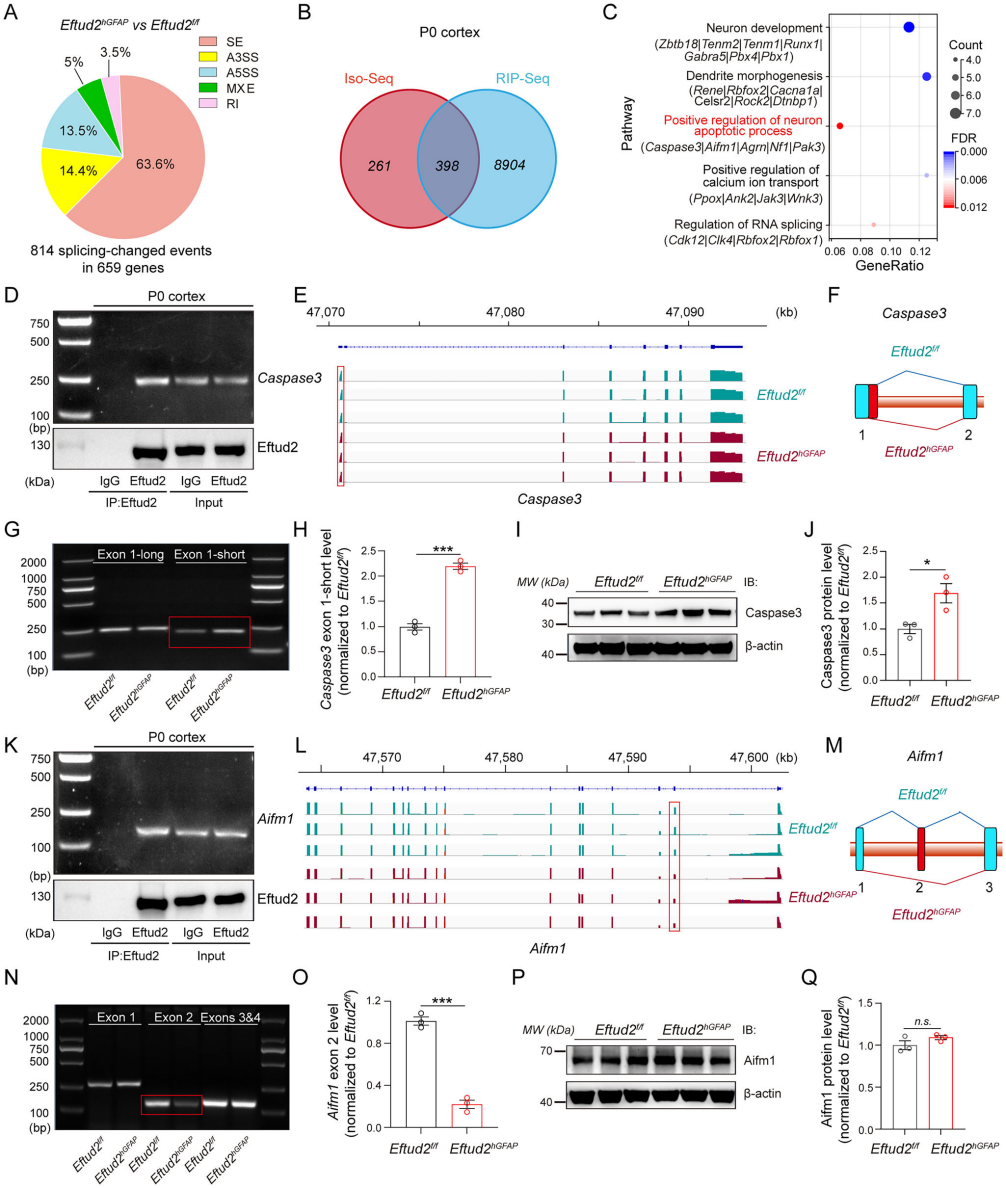
Eftud2 regulates apoptosis in NSCs and neurons via alternative splicing of *Caspase3* and *Aifm1*. A) Pie chart showing the proportion of alternative splicing events in the cortex of *Eftud2^hGFAP^* (cKO) mice. B) Venn diagram identifying 398 genes common to the Iso‐Seq gene set from *Eftud2^hGFAP^* cortex and the RIP‐seq (Eftud2‐IP) gene set from control (*Eftud2^f/f^*) cortex. C) GO pathway enrichment of the 398 shared genes reveals involvement in neuron development, dendrite morphogenesis, apoptosis, calcium ion transport, and RNA splicing. D) RNA immunoprecipitation (RIP) and co‐immunoprecipitation (co‐IP) confirm that Eftud2 interacts with *Caspase3* mRNA in brain cortex at P0. E) Integrative Genomics Viewer (IGV) tracks show exon expression changes in *Caspase3* in *Eftud2^hGFAP^* cortex. F) Schematic of *Caspase3* exon 1 A3SS in *GFAP‐Cre; Eftud2^f/f^* cKO mouse cortex. G, H) RT‐PCR (G) and quantification (H) confirm exon 1 A3SS in *Caspase3* in *Eftud2^hGFAP^* cortex (*n* = 3; unpaired *t*‐test). I, J) Immunoblot (I) and quantification (J) validate reduced Caspase3 protein levels in *Eftud2^hGFAP^* cortex (*n* = 3; unpaired *t*‐test). K) RIP and co‐IP validate that Eftud2 interacts with *Aifm1* mRNA and protein in control cortex at P0. L) IGV tracks show exon expression changes in *Aifm1* in *Eftud2^hGFAP^* cortex. M) Schematic of exon 2 skipping in *Aifm1* observed in *Eftud2^hGFAP^* cortex. N, Q) RT‐PCR (N) and quantification (O) confirm exon 2 skipping in *Aifm1* in cKO cortex (*n* = 3; unpaired *t*‐test). P, Q) Immunoblot (P) and quantification (Q) validate no change in Aifm1 protein levels in *Eftud2^hGFAP^* cortex (*n* = 3; unpaired *t*‐test). All data represent means ± SEM. *p < 0.05, ***p < 0.001, n.s., not significant.

EFTUD2, a core spliceosomal GTPase essential for spliceosome activation and splicing fidelity,^[^ ^31^, ^38^ ^]^ was investigated for its regulatory functions using full‐length transcriptome sequencing (Iso‐Seq). This high‐resolution methodology enables comprehensive reconstruction of full‐length mRNA architectures, including 5′/3′ untranslated regions (UTRs) and polyadenylation sites, without assembly‐dependent artifacts.^[^ ^39^ ^]^ It allows precise analysis of structural features such as alternative splicing and fusion genes within reference genomes. To examine changes in RNA alternative splicing in the cortex following *Eftud2* deletion, we employed Iso‐Seq to detect full‐length transcript variations. Splicing events analyzed included skipped exon (SE), retained intron (RI), alternative 5′ splice site (A5SS), alternative 3′ splice site (A3SS), and mutually exclusive exon (MXE), all of which are critical for cellular function and diversity.^[^ ^40^ ^]^ Cortical transcriptomes from P0 *Eftud2^f/f^* and *Eftud2^hGFAP^* mice were analyzed for splicing perturbations, revealing 659 genes with differential splicing patterns (false discovery rate [FDR] ≤ 5%), predominantly featuring SE (63.6%) over RI, A5SS/A3SS, or MXE (**Figure** **4**A).

To identify *Eftud2*'s functional targets, RNA immunoprecipitation (RIP) coupled with high‐throughput sequencing (RIP‐Seq) was performed on cortical lysates from *Eftud2^f/f^* mice, identifying direct RNA interactors. Intersectional analysis of RIP‐Seq targets with splicing‐altered genes in *Eftud2^hGFAP^* cortices revealed 398 overlapping loci (Figure 4B). GO enrichment analysis of these shared 398 targets demonstrated significant associations with neurodevelopmental pathways and apoptotic regulation (Figure 4C), with *Caspase3* and *Aifm1* emerging as top‐ranked apoptosis effectors. RIP assays confirmed the interactions between Eftud2 and the RNA transcripts of *Caspase3* and *Aifm1* in the cortices of *Eftud2^f/f^* mice (Figure 4D,K). Splicing architecture analysis using the Integrative Genomics Viewer (IGV) uncovered structural perturbations, including a 3′ splice site alteration in exon 1 of *Caspase3* and skipping of exon 2 in *Aifm1* (Figure 4E,L). Isoform‐resolved quantification further demonstrated increased shorter *Caspase3* exon 1 variants and exon 2‐skipping in *Aifm1* in the *Eftud2^hGFAP^* cortex (Figure 4F,M).

Reverse transcription polymerase chain reaction (RT‐PCR) assays confirmed a significant increase in the shorter *Caspase3* exon 1 isoform and a downregulation of *Aifm1* exon 2 expression in the *Eftud2^hGFAP^* cortex (Figure 4G,H,N,O). Additionally, Iso‐Seq analysis identified exon‐skipping events in *Pak3* exon 2, *Nf1* exon 12, and exons 34–35 of *Agrn* in the *Eftud2^hGFAP^* cortex (Figure S10A,D,G, Supporting Information). RT‐PCR analysis corroborated these exon‐skipping events (Figure S10B,C,E,F,H,I, Supporting Information), validating the fidelity of Iso‐Seq. Immunoblot analysis demonstrated that Caspase3 expression was significantly increased in *Eftud2^hGFAP^* mice, while Aifm1 level remained unchanged (Figure 4I,J,P,Q). Given the established apoptogenic roles of Caspase3 and Aifm1 in cells,^[^ ^41^, ^42^, ^43^, ^44^ ^]^ these findings mechanistically link EFTUD2 deficiency to apoptotic activation through splice‐directed generation of pro‐apoptotic *Caspase3* variants via aberrant 3′ splice site selection and truncated *Aifm1* isoforms through exon 2 skipping. This splice‐switching mechanism disrupts neurodevelopmental homeostasis by preferentially generating pro‐death molecular effectors.

To evaluate the conservation of splicing pathology across species, cortical NSC neurospheres were subjected to siRNA‐mediated *Eftud2* knockdown with concurrent lentiviral complementation (pGC‐FU‐3FLAG‐CBh‐gcGFP‐IRES‐puromycin vector expressing V5‐tagged human *EFTUD2* variants). Immunoblot validation confirmed efficient endogenous *Eftud2* depletion and ectopic protein expression (Figure S11A,B, Supporting Information). In agreement with in vivo observations (Figure 4), *Eftud2* knockdown in cortical NSC neurospheres significantly increased *Caspase3* short exon 1 and decreased *Aifm1* exon 2 (Figure S11E–J, Supporting Information). Co‐expression of wild‐type *EFTUD2* partially normalized these ratios, whereas the pathogenic missense mutants demonstrated variable corrective capacity for *Aifm1* and *Caspase3* exon splicing, while showing minimal rescue of *Aifm1* exon 2 splicing, all four missense mutants partially restored *Caspase3* short exon 1 levels, though not to the extent achieved by wild‐type EFTUD2 (Figure S11E–J, Supporting Information). These data confirm the evolutionary conservation of *EFTUD2*'s splice‐regulatory function in apoptotic activation.

To delineate the involvement of the Trp53 pathway, we quantified Trp53 expression in *Eftud2^hGFAP^* cortices through transcriptomic (RNA‐seq), quantitative PCR (qPCR), and immunoblot analyses. No significant upregulation of Trp53 was observed in *Eftud2^hGFAP^* mice (Figure S12, Supporting Information). The absence of Trp53 activation establishes that EFTUD2‐mediated apoptotic regulation operates through Trp53‐independent mechanisms in cortical development, contrasting with reported neural crest cell phenotypes. This mechanistic divergence underscores context‐dependent functionality of spliceosomal regulation in neurodevelopmental homeostasis.

### 2.5 Genetic Ablation of *Caspase3* Partially Rescues Corticogenic Deficits Induced by *Eftud2* Deficiency

**Figure 5:**
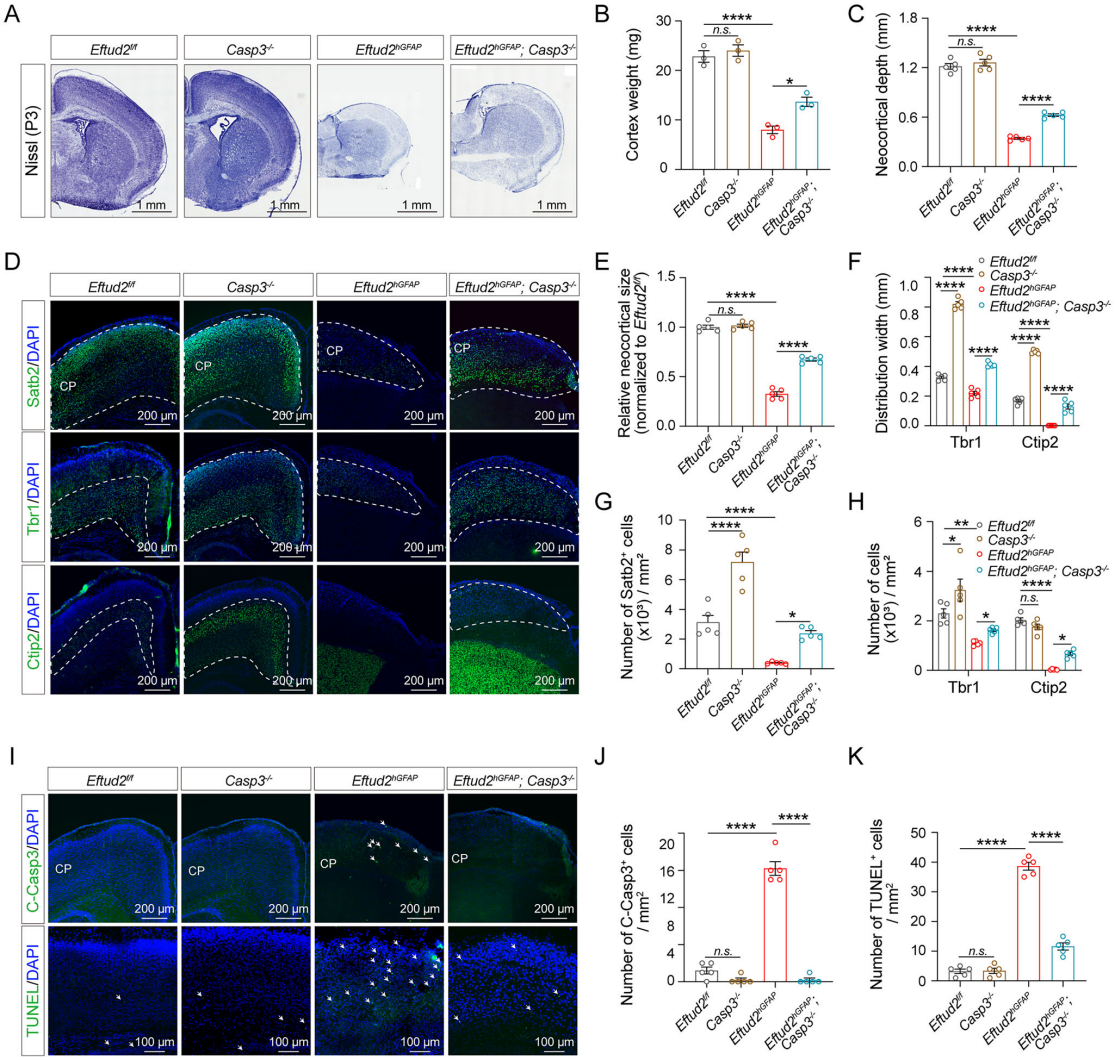
*Caspase3* co‐deletion partially rescues *Eftud2*‐deficiency phenotypes. A) Nissl‐stained coronal sections of *Eftud2^f/f^* (Control), *Caspase3^‐/‐^* (*Caspase3* KO, *Casp3^‐/‐^*), *Eftud2^hGFAP^* (*Eftud2* cKO), and *Eftud2^hGFAP^; Casp3^‐/‐^* (DKO) mice at P3 show thinner cortical layers in *Eftud2* cKO mice, which are partially restored in DKO mice. Scale bar: 1 mm. B, C) Quantification of cortical weight (B) and depth (C) indicates partial rescue in DKO mice compared to *Eftud2* cKO mice (*n* = 5 mice; one‐way ANOVA and Tukey multiple comparisons test). D) Immunofluorescence staining shows expression of the neocortical marker Satb2, deep‐layer markers Tbr1 (layer VI) and Ctip2 (layer V) in cortical sections. DKO mice exhibit improved cortical organization compared to *Eftud2* cKO mice. Scale bar: 200 µm. E‐H) Quantification of neocortical size (E), distribution width per unit area (F), Satb2^+^ cell numbers per unit area (G), and total cell numbers per unit area (H) shows significant recovery in DKO mice (*n* = 5 mice; one‐way or two‐way ANOVA and Tukey multiple comparisons test). I) Immunofluorescence staining for apoptosis markers C‐Casp3 (Top) and TUNEL (Bottom) in cortical sections. Apoptosis is significantly reduced in DKO mice compared to *Eftud2* cKO mice. Scale bars: 200 µm, 100 µm. J, K) Quantification of C‐Casp3^+^ cells (J) and TUNEL^+^ cells (K) in cortical sections reveals a marked reduction of apoptotic cells in DKO mice (*n* = 5 mice; one‐way ANOVA and Tukey multiple comparisons test). All data represent means ± SEM. *p < 0.05, **p < 0.01, ****p < 0.0001, n.s., not significant. Abbreviations: CP, cortical plate.

The 5′UTR‐localized exon 1 of *Caspase3* exhibits upregulated expression and alternative splicing in *Eftud2^hGFAP^* cortices, prompting investigation into its transcriptional regulatory role. To determine whether simultaneous deletion of *Caspase3* could rescue the neurogenic deficiencies observed in *Eftud2^hGFAP^* mice, we further generated *hGFAP‐Cre; Eftud2^f/f^*; *Casp3* ^‐/‐^ double‐knockout (*Eftud2^hGFAP^; Casp3^‐/‐^* DKO) mice by genetically ablating both *Eftud2* and *Caspase3* in the forebrain. Notably, *Caspase3* single knockout mice (*Casp3^‐/‐^*) exhibited no significant changes in brain length or cortical area, whereas *Eftud2^hGFAP^* mice demonstrated significant reductions in both metrics, as previously shown (Figure 2A–D; **Figure** **5**A,B). In *Eftud2^hGFAP^; Casp3^‐/‐^* mice, however, Nissl staining analysis showed that double deletion of *Caspase 3* and *Eftud2* produced improvements in cortical depth and weight at ≈50% of control levels (Figure 5A–C). These results indicate that while suppression of apoptosis partially mitigates the neurodevelopmental defects caused by Eftud2 deficiency, additional pathogenic mechanisms likely contribute to the observed abnormalities. Immunofluorescent staining revealed that *Eftud2^hGFAP^; Casp3^‐/‐^* mice exhibited a significant restoration of neocortical size, total Satb2^+^ cell numbers, and cortical lamination compared to *Eftud2* single mutants (Figure 5D–H). These results indicate that neuronal production in *Eftud2* mutants can be partially rescued by *Caspase3* deletion.

Furthermore, the increased apoptosis observed in *Eftud2^hGFAP^* mice was significantly mitigated by the simultaneous deletion of *Caspase3* in *Eftud2^hGFAP^; Casp3^‐/‐^* mice. Specifically, the number of apoptotic NSCs and neurons in *Eftud2* cKO mice was markedly reduced in *Eftud2^hGFAP^; Casp3^‐/‐^* mice (Figure 5I,J). TUNEL staining demonstrated a significant reduction in the number of TUNEL^+^ apoptotic cells within the cortex of *Eftud2^hGFAP^; Casp3^‐/‐^* mice at P3 compared to *Eftud2^hGFAP^* (Figure 5I,K). Together, these findings establish Caspase3‐mediated apoptosis as the predominant mechanism underlying *Eftud2* deficiency‐induced corticogenic failure. The partial phenotypic rescue implicates additional apoptotic effectors in MFDM pathology while confirming the crucial role of Caspase3 in spliceosomal dysregulation‐associated neurodevelopmental attrition.

### 2.6 Exon 2‐Deficient *Aifm1* Isoform Enhances Caspase3‐Mediated Apoptotic Cascades in Cortical Neural Populations

**Figure 6:**
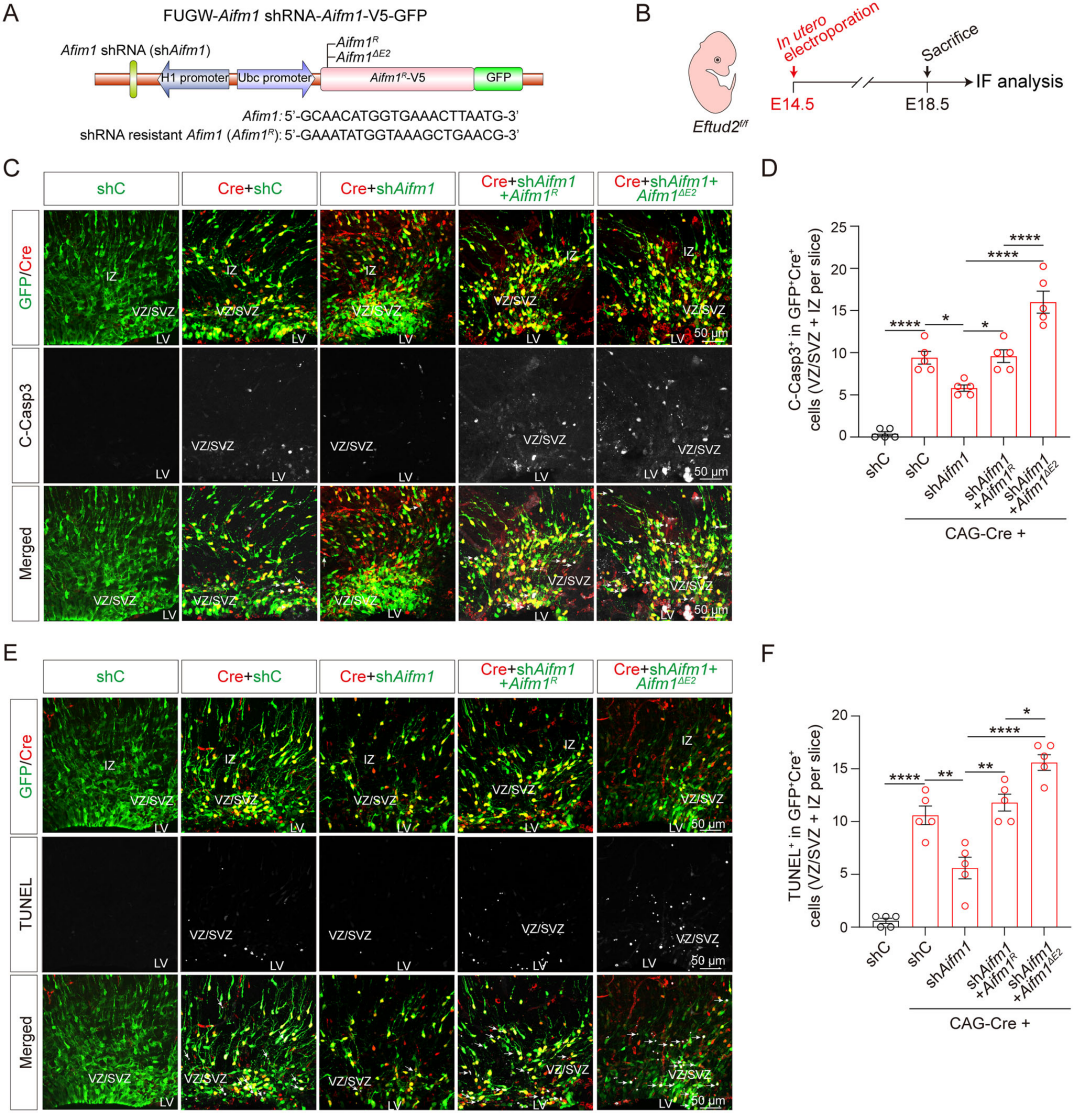
*Aifm1* exon 2 skipping potentiates Caspase3‐mediated apoptosis in the cortex. A) Schematic diagram of the Lenti‐FUGW‐*Aifm1* shRNA‐*Aifm1*‐V5‐GFP plasmid, designed for endogenous mouse *Aifm1* knockdown and ectopic expression of exogenous shRNA‐resistant mouse *Aifm1* (*Aifm1^R^*) or *Aifm1* exon 2 skipping mutant (*Aifm1^ΔE2^*). Four constructs were used: shRNA control (shC), *Aifm1* shRNA knockdown (sh*Aifm1*), *Aifm1* shRNA knockdown and ectopic expression of shRNA‐resistant mouse *Aifm1* (sh*Aifm1* + *Aifm1^R^*) or shRNA‐resistant *Aifm1* exon 2 skipping truncated mutant (sh*Aifm1* + *Aifm1^ΔE2^*). B) Schematic diagram of in utero electroporation and experiment timeline and procedure for panels C‐F. C) Co‐localization assay of C‐Casp3^+^ apoptotic cells with Cre^+^ cells and GFP^+^ electroporated cells within the VZ/SVZ and IZ following in utero electroporation. Scale bar: 50 µm. D) Quantification of C‐Casp3^+^Cre^+^GFP^+^ triple positive cells within the VZ/SVZ and IZ (*n* = 5 mice; one‐way ANOVA and Tukey multiple comparisons test). E) Co‐localization assay of TUNEL^+^ apoptotic cells with Cre^+^GFP^+^ double positive electroporated cells within the VZ/SVZ and IZ following in utero electroporation. Scale bar: 50 µm. F) Quantification of TUNEL^+^Cre^+^GFP^+^ triple positive cells within the VZ/SVZ and IZ (*n* = 5 mice; one‐way ANOVA and Tukey multiple comparisons test). All data represent means ± SEM. *p < 0.05, **p < 0.01, ****p < 0.0001. Abbreviations: LV, lateral ventricle, VZ, ventricular zone; SVZ, subventricular zone; IZ, intermediate zone.

Given Aifm1's established role in Caspase3 activation pathways,^[^ ^45^ ^]^ we employed a combinatorial genetic strategy to delineate its contribution to corticogenic apoptosis. Using the FUGW vector,^[^ ^46^, ^47^ ^]^ we constructed H1 promoter‐driven shRNA for endogenous *Aifm1* knockdown, along with Ubc promoter‐driven overexpression constructs for either wild‐type *Aifm1* (*Aifm1^R^*) or exon 2‐deleted Aifm1 (*Aifm1^ΔE2^*), both of which were resistant to *Aifm1*‐targeting shRNA (**Figure** **6**A). Caspase3 activation analysis revealed a significant reduction in cleaved‐Caspase3 levels in the shAifm1‐treated group compared to the control group expressing non‐targeting shRNA (shC) in P19 cells (Figure S13A–C, Supporting Information). Notably, within the sh*Aifm1*‐treated samples, cells expressing *Aifm1^ΔE2^* exhibited markedly higher levels of cleaved‐Caspase3 compared to those expressing *Aifm1^R^* (Figure S13A–C, Supporting Information).

To determine whether exon 2 skipping in *Aifm1* contributes to apoptosis activation and neuronal loss, we performed in utero electroporation assays in *Eftud2^f/f^* cortices (Figure 6B). Knockdown of *Aifm1* using sh*Aifm1* reduced apoptosis in NSCs and neurons induced by *Eftud2* loss in *Eftud2^f/f^* mice expressing Cre recombinase (Figure 6C–F). However, following Cre‐mediated *Eftud2* knockout, reintroduction of *Aifm1^ΔE2^* significantly enhanced Caspase3 activation and increased the number of cleaved‐Caspase3^+^ or TUNEL^+^ cells in NSCs and neurons within the cortex at E18.5 compared to those expressing *Aifm1^R^* (Figure 6C–F). These findings suggest that exon 2 skipping in *Aifm1* is critical for apoptosis activation in NSCs and neurons.

Additionally, we assessed NSC apoptosis in vitro using primary NSC cultures (Figure S14A, Supporting Information). Lentiviral vectors were generated for four experimental groups: shC, sh*Aifm1*, sh*Aifm1* combined with wild‐type *Aifm1* (sh*Aifm1* + *Aifm1^R^*), and sh*Aifm1* combined with exon 2‐deleted *Aifm1* (sh*Aifm1* + *Aifm1^ΔE2^*). Primary NSCs were first transfected with non‐specific Scramble siRNA (Scramble) or specific siRNA targeting *Eftud2* (si*Eftud2*). Twelve hours post‐transfection, cells were infected with the four lentiviral constructs. Six days postlentiviral infection, several protein levels and apoptosis were assessed. Consistent with observations in P19 cells, exon 2 skipping of *Aifm1* in NSC neurospheres increased C‐Casp3 levels (Figure S13D–F, Supporting Information). Knockdown of *Eftud2* alone induced apoptosis in NSCs, as evidenced by increased C‐Casp3^+^ or TUNEL^+^ cell ratios. However, knockdown of endogenous *Aifm1* reduced these apoptosis markers caused by *Eftud2* knockdown (Figure S14B–E, Supporting Information). Interestingly, the si*Eftud2* + sh*Aifm1* + *Aifm1^ΔE2^* group exhibited a substantial increase in C‐Casp3^+^ and TUNEL^+^ cells compared to the si*Eftud2* + sh*Aifm1* + *Aifm1^R^* group (Figure S14B–E, Supporting Information). Furthermore, reducing endogenous *Aifm1* levels increased the diameter of NSC neurospheres compared to the si*Eftud2* group, confirming the role of Aifm1 in mediating *Eftud2* deficiency‐induced apoptosis (Figure S14F,G, Supporting Information). In contrast, overexpression of truncated *Aifm1^ΔE2^* significantly decreased neurosphere diameter compared to overexpression of *Aifm1^R^* (Figure S14F,G, Supporting Information).

In summary, these findings demonstrate that exon 2 exclusion in *Aifm1* generates pro‐apoptotic isoforms which synergistically amplify Caspase3 activation, driving neurodevelopmental attrition in *Eftud2*‐deficient cortices. The conserved in vivo and in vitro phenotypes highlight a splicing‐dependent mechanism through which *Aifm1* alternative splicing regulates apoptotic homeostasis during cortical morphogenesis.

## 3 Discussion

This study elucidates the molecular pathogenesis underlying MFDM by establishing a direct causal relationship between *EFTUD2* dysfunction, aberrant RNA splicing fidelity, and corticogenesis failure. Our findings demonstrate that both human pathogenic *EFTUD2* mutations and conditional *Eftud2* deletion disrupt the splicing landscape of apoptotic regulators *Caspase3* and *Aifm1*, driving cortical neuronal attrition through isoform‐specific activation of programmed cell death. The observed reduction in neuronal density and cortical thinning in *Eftud2^hGFAP^* mice (Figure 2G–I) faithfully recapitulate the neurodevelopmental hallmarks of MFDM, positioning *EFTUD2* as a critical guardian of spliceosomal homeostasis during corticogenesis. These results extend beyond previous associations of *EFTUD2* with craniofacial development by mechanistically linking spliceosomal dysfunction to cerebral cortical malformations.

The precise orchestration of alternative splicing during cortical development requires dynamic coordination between U5 small nuclear ribonucleoprotein (snRNP) components and auxiliary RNA‐binding proteins that integrate epigenetic and environmental cues.^[^ ^48^, ^49^, ^50^ ^]^ Disruptions in these splicing events can lead to abnormal cortical development, resulting in structural and functional brain abnormalities.^[^ ^51^, ^52^ ^]^ Consequently, understanding the molecular mechanisms governing splicing regulation in cortical development is essential for identifying potential therapeutic targets for neurodevelopmental disorders.^[^ ^53^ ^]^ Our data reveal that *Eftud2* deficiency disrupts this equilibrium through two synergistic pathways: generation of pro‐apoptotic *Aifm1^ΔE2^* isoforms and *Caspase3* variants with altered 3' splice sites in exon 1. The conserved neuropathology across NSC‐specific (*Eftud2^hGFAP^*) and cortico‐specific (*Eftud2^Emx1^*) models underscores *Eftud2*’s non‐redundant role in maintaining progenitor pool integrity, suggesting evolutionary conservation of its spliceoregulatory functions in mammalian neurodevelopment. These findings offer key insights into the etiology of neurodevelopmental disorders associated with *EFTUD2* mutations and the dynamics of RNA splicing and RNA‐binding protein (RBP) expression.

In the nervous system, Eftud2 is crucial for the survival of neural precursor cells in zebrafish and neural crest cells and Purkinje cells in mice.^[^ ^16^, ^22^, ^24^ ^]^ In addition, Eftud2 in mouse microglia regulates activation and polarization through the NF‐κB signaling pathway.^[^ ^54^ ^]^ However, the exact role and its underlying mechanisms of EFTUD2 in the development and functional maintenance of the cerebellar cortex remains poorly understood. In the cerebellar cortex, loss of *Eftud2* induces apoptosis in NSCs and neurons by mediating the alternative splicing of apoptotic genes, consistent with observations in neural crest cells.^[^ ^16^ ^]^ Interestingly, in Purkinje cells, *Eftud2* deficiency leads to neuronal degeneration via ferroptosis, rather than Trp53‐dependent apoptosis.^[^ ^24^ ^]^ Unlike its effects in Purkinje cells, *Eftud2* ablation does not significantly alter the expression of ferroptosis‐related genes in the cerebellar cortex, suggesting that Eftud2 regulates neurogenesis and neuronal differentiation through distinct mechanisms depending on the cell type and cell lineage. These findings highlight the diverse roles of Eftud2 in neuronal survival and differentiation in mammals.

While our findings identify *Caspase3* and *Aifm1* as functional targets of Eftud2 in the mouse cortex, it remains possible that Eftud2 regulates the alternative splicing of other genes involved in the apoptosis signaling pathway. Iso‐seq analysis further revealed alternative splicing events in additional genes associated with apoptosis, such as *Pak3*, *Nf1*, and *Kif7*, all showing substantial exon skipping (Figure S10, Supporting Information). Although these genes do not directly interact with Eftud2, their spliced isoforms may play significant roles in cerebellar cortex development. Interestingly, the alternative splicing of *Mdm2* was not detected in the cortex of *Eftud2^hGFAP^* cKO mice compared to *Eftud2^f/f^* mice, which contrasts with previous findings.^[^ ^16^ ^]^ This discrepancy may be due to differences in cell types, sequencing technologies, or the specific molecular mechanisms underlying apoptotic activation induced by *Eftud2* deletion in distinct cells. Additionally, alternative splicing of genes involved in neuronal development, such as *Zbtb18*, *Tenm2*, and *Tenm1*, may also contribute to cortical developmental defects following *Eftud2* loss‐of‐function. Further investigation is needed to determine whether a mutual regulatory relationship exists between the RNA splicing events of key apoptosis regulators and neuronal development in cortical formation.

These findings may have significant implications for understanding the pathogenesis of MFDM in humans. By linking *EFTUD2* mutations to splicing defects in apoptosis‐related genes, this study provides a unifying mechanism for the neuronal loss and cortical abnormalities characteristic of this disorder. Furthermore, these results suggest that targeting alternative splicing could be a potential therapeutic strategy for MFDM. Approaches such as antisense oligonucleotides (ASOs) and small molecules that modulate splicing have shown promise in treating other genetic disorders, such as spinal muscular atrophy (SMA).^[^ ^55^, ^56^, ^57^ ^]^ Similar strategies may be explored to correct the splicing defects in *CASPASE3* and *AIFM1* caused by *EFTUD2* mutations in patients. Additionally, targeting downstream apoptotic pathways, such as Caspase3 inhibition, may offer a complementary approach to mitigate neuronal loss in MFDM.

Future investigations should address critical unresolved questions, including the temporal requirements for splicing correction, potential compensatory mechanisms involving alternative spliceosomal components, and network‐level crosstalk between apoptotic and neurodevelopmental splicing events. By mechanistically linking *EFTUD2* mutations to a unified pathway of splicing‐dependent apoptosis, this work repositions MFDM within the emerging class of spliceosomopathies and establishes a framework for targeted therapeutic development in neurodevelopmental disorders.

## 4 Experimental Section

### Mouse Strains and Genotyping

*hGFAP*‐Cre (JAX: 004600), *Emx1*‐Cre (JAX: 005628), and *Caspase3*‐deficient (*Casp3^‐/‐^*) (*Caspase3^tm1Flv^*, JAX: 006233) mice were maintained on a C57BL/6 genetic background under specific pathogen‐free conditions.^[^ ^35^, ^36^, ^37^, ^58^, ^59^ ^]^ *Eftud2* floxed (*Eftud2^f/f^*) mice were kindly provided by Dr. Guojiang Chen (Beijing Institute of Pharmacology and Toxicology), and the *Casp3^‐/‐^* mice were gifted by Dr. Shengxi Wu (Fourth Military Medical University). Conditional knockout mice (*Eftud2^hGFAP^* and *Eftud2^Emx1^*) were generated by crossing *Eftud2^f/f^* mice with Cre‐driver lines. Littermate *Eftud2^f/f^* mice lacking Cre recombinase served as controls. All mice were bred between 8 and 12 weeks of age, with both male and female mice selected randomly for experiments. The ages and developmental stages of the mice are indicated in the figures and figure legends.

Brains used for phenotypic analyses were obtained from mice bred for more than five generations on a C57BL/6 background. The day a vaginal plug was observed was designated as embryonic day 0.5 (E0.5), and the day of birth was considered postnatal day 0 (P0). Animals were housed under a 12:12 light‐dark cycle with ad libitum access to food and water.

All animal experiments were conducted in accordance with protocols approved by the Institutional Animal Care and Use Committee of the Beijing Institute of Basic Medical Sciences (protocol #SYXK2019‐0004). Details of genotyping sequences are provided in Table S1 (Supporting Information).

### In Utero Electroporation

In utero electroporation was performed on *Eftud2^f/f^* embryonic mice at E14.5 as previously described.^[^ ^60^ ^]^ Pregnant *Eftud2^f/f^* mice were anesthetized with an intraperitoneal injection of 0.1% sodium pentobarbital (6 g kg^−1^). The uterine horn was gently exposed, and the lateral ventricles of embryos were injected with 1 µL of DNA solution mixed with Fast Green (0.1 mg mL^−1^, Sigma–Aldrich) using a pulled micropipette.

For experiments involving *Eftud2* knockout and rescue by human *EFTUD2* or its mutants, the following DNA constructs were injected into the lateral ventricles of E14.5 *Eftud2^f/f^* embryos: pCMS‐EGFP control (1 µg µL^−1^), pCAG‐mCherry‐Cre (1 µg µL^−1^), or pCAG‐mCherry‐Cre (1 µg µL^−1^) combined with *EFTUD2* or its mutant constructs. For experiments assessing *Eftud2* knockout and overexpression of GFP‐tagged wild‐type *EFTUD2* or *EFTUD2* mutants (R262W, C476R, L637R, A823T), the constructs were injected at a 1:1 ratio into E14.5 *Eftud2^f/f^* embryos.

For studies of alternative RNA splicing in *Aifm1* in NSCs and neurons, the following DNA constructs were injected into the lateral ventricles of E14.5 *Eftud2^f/f^* embryos: FUGW‐GFP control (shC) (1 µg µL^−1^), pCAG‐mCherry‐Cre (1 µg µL^−1^) combined with FUGW‐GFP vector, or pCAG‐mCherry‐Cre (1 µg µL^−1^) combined with FUGW‐sh*Aifm1*‐GFP (1 µg µL^−1^) or FUGW‐sh*Aifm1*‐*Aifm1^R^* (1 µg µL^−1^), FUGW‐sh*Aifm1*‐*Aifm1^ΔE2^* (1 µg µL^−1^) constructs.

Electroporation was conducted using an ECM 830 electroporator (BTX) with five 50 ms pulses separated by 950 ms intervals at 40 V. Each DNA pool was injected into three to four embryos in at least two independent experiments. Following electroporation, the uterine horns were repositioned, and the abdominal cavity was bathed with pre‐warmed sterile saline. The surgical incision was sutured, and the mice were monitored on a heating pad until fully recovered, after which they were returned to their home cage and observed for signs of pain or distress. Embryos were harvested at E18.5 or postnatal stages (P7/P14) for analysis. Coronal sections (25 µm) were imaged on an FV‐1200 confocal microscope (Olympus), with three non‐adjacent sections per brain quantified using ImageJ (NIH) and Adobe Photoshop CS (Adobe Systems).

### Cell Culture, Plasmid Transfection

HEK293T cells (ATCC, CRL‐11268™) and P19 mouse embryonal carcinoma cells (ATCC, CRL‐1825) were maintained in Dulbecco's Modified Eagle Medium (DMEM, Gibco, C11995500BT) supplemented with 10% fetal bovine serum (FBS, Gibco, 10091148). Cells were maintained at 37 °C in a humidified atmosphere with 5% CO_2_. For transfection, HEK293T or P19 cells were transfected with plasmids using Lipofectamine 2000 (Invitrogen, 11668019) following the manufacturer's protocol. Transfection was carried out for 48 h before subsequent analysis.

### Immunofluorescence Staining and Image Processing

Mice were anesthetized with 1% sodium pentobarbital (6 g kg^−1^, Sigma–Aldrich, 52944‐66‐8) and perfused transcardially with 0.9% NaCl, followed by 4% paraformaldehyde (PFA, Solarbio, P1110). Brains were removed, post‐fixed overnight at 4 °C, cryoprotected in 15% and 30% sucrose (Sigma–Aldrich, 57‐50‐1) sequentially for 48 h, and embedded in optimal cutting temperature (O.C.T.) compound (SAKURA, #4583) before cryosection. Sagittal brain sections (20–40 µm) were obtained using a cryostat, mounted on slides, and air‐dried for 30 min at room temperature (RT).

Sections or primary spheres were rehydrated in phosphate‐buffered saline (PBS) for 15 min, washed in PBST (PBS + 1% Triton X‐100) for 30 min at RT, and blocked with 3% bovine serum albumin (BSA) in PBST (PBS + 0.3% Triton X‐100) for 1 h at RT. Samples were then incubated overnight at 4 °C with primary antibodies diluted in 3% BSA/PBST. The following primary antibodies and dilutions were used: anti‐Eftud2 (Abcam, ab188327, 1:400), anti‐Satb2 (Abcam, ab92446, 1:400), anti‐Tbr1 (Thermo Fisher Scientific, ab104224, 1:200), anti‐Ctrip2 (Abcam, ab92446, 1:400), anti‐CUX1 (Sigma‐Aldrich, C9848, 1:200), anti‐Sox2 (Abcam, ab79351, 1:400), anti‐NeuN (Abcam, ab104224, 1:800), anti‐Cre (Sigma, Mab3120, 1:400), anti‐GFP (Invitrogen, A‐11122, 1:500), anti‐mCherry (BD Bioscience, ab125096, 1:400), anti‐V5 (Invitrogen, MA5‐15253, 1:400), and anti‐Cleaved‐Caspase‐3 (Cell Signaling Technology, 9661, 1:400), anti‐EGFR (Abmart, T55112F, 1:300).

After three washes in PBS (5 min each), sections were incubated with Alexa Fluor 568‐, Alexa Fluor 488‐or Alexa Fluor 633‐conjugated secondary antibodies (Biotium, 20012, 20101, 20102, 20018, 20124, 20125) diluted in PBST + 3% BSA (1:500) for 3 h at RT. Sections were washed in PBS, counterstained with DAPI (ZSGB‐BIO, ZLI‐9557), and mounted using fluorescent mounting medium. Images were captured using an Olympus FV‐1200 confocal microscope (Tokyo, Japan).

### Nissl Staining

Brain slices were washed three times with PBS for 5 min each. Subsequently, the slices were immersed in 0.5% cresyl violet solution (Beyotime, C0117) for 20 min. After staining, the slices were briefly rinsed briefly in distilled water to remove excess dye. Differentiation was performed by incubating the slices in 95% ethanol for 2 min. The slices were then sequentially dehydrated in 75%, 90%, and 100% ethanol for 30 s each. Finally, the slices were mounted using neutral resin and prepared for analysis.

### Immunoprecipitation and Immunoblotting

Mouse cortex, primary NSC spheres, HEK293T cells and P19 cells were lysed in buffer containing 50 mm Tris (pH 7.5), 150 mm NaCl, 10 mm MgCl₂, 1% Triton X‐100, 10% glycerol, 1 mm EDTA, 1 mm PMSF (Beyotime, ST507), and 1% protease inhibitors (Thermo Fisher Scientific, A32965). Lysates were centrifuged at 12 000 rpm for 15 min at 4 °C. For immunoprecipitation, protein lysates were incubated with primary antibodies and protein A/G magnetic agarose beads (Thermo Scientific, 20423). Immunocomplexes were washed six times with washing buffer (50 mm Tris‐HCl, pH 7.5, 150 mm NaCl, 1 mm EDTA, 1% Triton X‐100) and analyzed by immunoblotting.

For immunoblotting, protein supernatants were mixed with loading buffer and boiled for 5 min. The samples were separated on SDS‐PAGE gels, and transferred onto polyvinylidene fluoride (PVDF) membranes (Millipore, IPFL00010). The membranes were blocked with PBST (PBS containing 0.1% Tween‐20) supplemented with 5% fat‐free milk for 1 h at room temperature. After blocking, the membranes were incubated with primary antibodies overnight at 4 °C, followed by incubation with horseradish peroxidase (HRP)‐conjugated secondary antibodies for 2 h at room temperature. Protein bands were visualized using enhanced chemiluminescence (ECL) reagents (Applygen Technologies, Beijing, China) and imaged with an Alpha Innotech FluorChemQ MultiImage III system. Primary antibodies used for Western blot and co‐immunoprecipitation are as follows: anti‐Eftud2 (Abcam, ab72456, 1:1000), anti‐V5 (Invitrogen, MA5‐15253, 1:400), anti‐Cleaved‐Caspase‐3 (Cell Signaling Technology, 9661, 1:1000), anti‐Aifm1 (ABclonal, A0811, 1:1000), anti‐Caspase‐3 (Cell Signaling Technology, # 9662S, 1:1000), anti‐Trp53 (Abcam, ab32049, 1:3000), anti‐GFP (Invitrogen, A‐11122, 1:3000), anti‐HA (Sungene Biotech, KM8004, 1:2000), and anti‐β‐actin (Sungene Biotech, KM9001T, 1:3000).

### Plasmid Construction and Lentiviral Packaging

The human *EFTUD2* expression plasmid, pCEFL3XhaEFTUD2 was reorganized to incorporate the pCMS‐EGFP vector via PCR. Mutants (R262W, C476R, L637R, and A823T) were generated using the Mut Express MultiS Fast Mutagenesis Kit V2 (Vazyme, C215) with primers listed in Table S1 (Supporting Information). PCR products were digested with DpnI to remove methylated template DNA and transformed into *E. coli* DMT chemically competent cells (Transgen, CD511). Single clones were selected after 12 h and verified by sequencing.

For endogenous *Aifm1* deletion and exogenous overexpression, H1‐shRNA sequences targeting *Aifm1* and mouse *Aifm1* cDNA were synthesized (RuiBiotech, Beijing) as detailed in Table S1 (Supporting Information). A 288 bp fragment of H1‐*Aifm1*‐shRNA was subcloned into the PacI‐digested FUGW vector^[^ ^47^ ^]^ (provided by Dr. Zilong Qiu, Songjiang Research Institute, Shanghai Jiaotong University School of Medcine) to construct FUGW‐*Aifm1*‐shRNA. The orientation of the H1*‐Aifm1*‐shRNA fragment was confirmed by sequencing. For *Aifm1* overexpression, six silent mutations were introduced into pCMS‐*Aifm1*‐WT (*Aifm1^R^*) to reduce shRNA knockdown without altering the amino acid sequence. The exon 2 skipping mutant (*Aifm1^ΔE2^*) was created by mutating the exon sequence into alanine using the same mutagenesis kit. DNA segments of *Aifm1^R^* and *Aifm1^ΔE2^* were amplified, purified, transformed into *E. coli* DH5α, and sequenced. Construction of FUGW‐shRNA‐*Aifm1^R^* and FUGW‐shRNA‐*Aifm1^ΔE2^* followed similar procedures.

Splicing reporter vectors, pcDNA3.1‐Luci and pcDNA3.1‐Luci‐I, were constructed as described previously.^[^ ^27^, ^28^ ^]^ Wild‐type luciferase (Luci‐WT) and luciferase‐intron (Luci‐I) sequences with CL1 and PEST tags were synthesized (Ruibiotech) and subcloned into the pcDNA3.1(+) vector (BamHI/EcoRI). In pcDNA‐Luci‐I, an immunoglobulin intron (Promega, #U47119) was inserted into the luciferase gene. The pKH3‐*PRPF8* plasmid was constructed by amplifying the full‐length *PRPF8* coding sequence (CDS) via PCR and inserting it into the pKH3 vector (XhoI/NotI) using the ClonExpress Ultra One Step Cloning Kit (Vazyme, C115).

For lentiviral packaging, FUGW‐shC, FUGW‐sh*Aifm1*, FUGW‐sh*Aifm1*+*Aifm1^R^*, and FUGW‐sh*Aifm1*+*Aifm1^ΔE2^* plasmids were co‐transfected with VSVG and psPAX2 into HEK293T cells at a 5:3:2 ratio. Viral supernatants were collected at 36 and 72 h, centrifuged at 4000 rpm (4 °C), filtered through a 0.45 µm membrane, and ultracentrifuged at 80 000 × g for 2 h (4 °C). Lentiviral pellets were resuspended in DMEM/F‐12 (Gibco, 11330500). Viral titers were determined by real‐time quantitative PCR. For lentiviral transduction, primary NSC neurospheres were infected with lentiviruses carrying FUGW‐shC, FUGW‐sh*Aifm1*, FUGW‐sh*Aifm1*+*Aifm1^R^*, FUGW‐sh*Aifm1*+*Aifm1^ΔE2^*, pGC‐FU‐GFP, pGC‐FU‐*EFTUD2*, and pGC‐FU‐*EFTUD2* mutants (R262W, C476R, L637R, A823T) (titer: 1 × 10^8^ TU/mL, 10 µL) in culture medium, 6 h after siRNA transfection. Lentiviral particles were purchased from GENEchem (Shanghai, China).

Short interfering RNAs (siRNAs) targeting *Eftud2* and scramble control siRNA were synthesized by Ribobio (Guangzhou, China). The siRNA sequences targeting *Eftud2* are listed in Table S1 (Supporting Information). These siRNAs were transfected into cells using Lipofectamine™ RNAiMAX (Invitrogen, 13778150) according to the manufacturer's instructions.

### Luciferase Assay

To evaluate the effect of *EFTUD2* point mutations on intron splicing efficiency, HEK293T cells were transfected with expression plasmids for human *EFTUD2* (WT or mutants), the pRL‐TK luciferase reporter as an internal reference, and either pcDNA3.1‐Luc or pcDNA3.1‐Intron luciferase reporter plasmids using Lipofectamine 2000 (Invitrogen, 11668019). Empty pCMS plasmid was included to ensure equal DNA amounts across transfections.

To verify the endogenous splicing efficiency of *EFTUD2*, HEK293T cells were transfected with negative control (Scamble) or *EFTUD2* siRNA (RiboBio, Guangzhou) using Lipofectamine™ RNAiMAX (Invitrogen, 13778100). After 12 h, pcDNA3.1‐Luc, pcDNA3.1‐Intron, and pRL‐TK plasmids were introduced. Cells were harvested 48 h post‐transfection. Luciferase activity was measured using the Dual‐Luciferase Reporter Assay System (Promega, E1910) following the manufacturer's instructions. Firefly luciferase activity was normalized to Renilla luciferase activity to account for variations in transfection efficiency, as previously described.^[^ ^28^, ^46^, ^61^ ^]^

### RNA Extraction, qRT‐PCR, and RT‐PCR Analysis

Total RNA was extracted from mouse cortical tissues using TRIzon Reagent (CWBio, CW0580). One microgram of RNA from each sample was reverse transcribed into cDNA with HiScript III All‐in‐one RT SuperMix (Vazyme, R333‐01). Quantitative real‐time PCR (qRT‐PCR) was performed using the UltraSYBR One‐Step RT‐qPCR Kit (CWBio, CW0659), and reverse transcription PCR (RT‐PCR) was conducted with the 2× Phanta Flash Master Mix (Vazyme, P520‐01). Gene expression levels were quantified by averaging triplicate measurements across three independent experiments. PCR primers were synthesized by RuiBiotech (Beijing, China), and their sequences are listed in Table S1 (Supporting Information).

### RNA‐Binding Protein Immunoprecipitation and Sequencing (RIP‐Seq)

RNA‐binding protein immunoprecipitation (RIP) was performed using the RIP Assay Kit (MBL, RN1001) according to the manufacturer's instructions. RNA‐protein complexes were immunoprecipitated using an anti‐Eftud2 antibody (Novus, NBP2‐92930). Total RNA was extracted from the immunoprecipitated complexes and subjected to sequenced on the Illumina Novaseq platform by Annoroad Gene Technology (Beijing, China). RT‐PCR analysis of the resulting data was conducted using primers listed in Table S1 (Supporting Information).

### RNA‐Sequencing, Isoform Sequencing, and Bioinformatics Analysis

Total RNA was extracted from mouse cortical tissue using TRIzon Reagent (CWBio, CW0580), and RNA quality and concentration were assessed with a NanoDrop spectrophotometer (Thermo Fisher Scientific). High‐throughput RNA sequencing (RNA‐seq) was performed on the Illumina Novaseq 6000 platform by BerryGenomics (Beijing, China). To optimize the balance between analytical sensitivity and specificity in differential gene expression analysis, a log2Fold change cutoff of 0.25 (FC≈1.2) was set for preliminary screening. Datasets of genes' log₂FC values with adjusted *p*‐values and GO‐enriched pathways' adjusted *p*‐values are listed in Tables S2 and S3 (Supporting Information), respectively. Isoform sequencing (Iso‐seq) libraries were prepared using the PacBio Sequel platform (BerryGenomics, Beijing, China).

Gene ontology (GO) enrichment analysis was performed using clusterProfiler (version 3.10.0), DAVID bioinformatics resources (https://david.ncifcrf.gov/), and KOBAS bioinformatics resources (http://bioinfo.org/kobas). This integrated approach facilitated the identification of key pathways associated with the datasets.

### GTPase Activity Assay

The GTPase activity of wild‐type (WT) and mutant V5‐EFTUD2 proteins was measured using the GTPase Activity Assay Kit (MAK113, Sigma–Aldrich). V5‐EFTUD2 proteins were immunoprecipitated from HEK293T cells overexpressing WT or mutant *EFTUD2* constructs using a V5 antibody and Protein A/G magnetic beads. The immunoprecipitates were then subjected to GTPase activity detection following the kit's instructions and previously established protocols.^[^ ^62^ ^]^

### Prediction of 3D Protein Structure

The 3D structure of EFTUD2 was predicted using the crystallized EFTUD2 structure as a template. The wild‐type and mutant EFTUD2 structures were generated by importing the respective amino acid sequences into PyMOL software (PyMOL Molecular Graphics System, Version 1.2r3pre, Schrödinger, LLC). Differences in predicted hydrogen bond formation between the wild‐type and mutant structures were compared and visualized.

### NSC Neurosphere Isolation, siRNA Treatment, and Lentivirus Infection

Primary NSCs were isolated from the cortex of E12.5 *Eftud2^f/f^* mice, according to the previously published work.^[^ ^60^, ^63^ ^]^ Briefly, the cortex was dissected in primary medium [DMEM/F‐12 containing 2% B27 (Gibco, 17504044), 20 ng mL^−1^ recombinant human epidermal growth factor (EGF) (Gibco, PHG0311L), and 20 ng mL^−1^ basic fibroblast growth factor (bFGF) (Gibco, 13256029)] on ice. After removing the meninges, the cortical tissue was chopped into small pieces and incubated with a mixture of primary medium and 0.25% trypsin (Gibco, 25200056) (v/v, 1:1) at 37 °C for 30 min. The trypsin reaction was stopped by adding 10% fetal bovine serum (FBS), and the mixture was centrifuged at 1000 rpm for 5 min. The cells were rinsed with primary medium and filtered through a 70 µm cell strainer. The NSCs were gently dispersed into a single‐cell suspension by pipetting and centrifuged again at 1000 rpm for 5 min. The pelleted cells were resuspended in primary medium and seeded at a density of 1–2 × 10^6^ cells in T12.5 cell culture flasks, followed by incubation at 37 °C and 5% CO_2_.

One hour after seeding, siRNAs targeting *Eftud2* and scramble control siRNA were transfected into cells using Lipofectamine™ RNAiMAX (Invitrogen, 13778150) according to the manufacturer's instructions. For lentiviral infection, viruses containing FUGW‐shC, FUGW‐sh*Aifm1*, FUGW‐sh*Aifm1*+*Aifm1^R^*, FUGW‐sh*Aifm1*+*Aifm1^ΔE2^* (Titer: 1 x 10^8^ TU/mL, 10 µL) were added to the medium 5 h after seeding the primary NSCs.

### Data and Materials Availability

Original RNA‐seq, ISO‐seq, and RIP‐seq data have been deposited in the National Center for Biotechnology Information Sequence Read Archive (PRJNA1232059).

### Quantification and Statistical Analysis

All experiments were performed in at least triplicate, and data are presented as means ± SEM. Statistical analyses, including unpaired *t*‐tests, one‐way or two‐way ANOVA, and multiple comparisons tests, were conducted using GraphPad Prism 9 and SPSS. Image processing and analysis were performed using ImageJ (NIH) and NDP.View2 software (Hamamatsu). Statistical significance was defined as *p* < 0.05 (significant) and *p* < 0.01 (highly significant).

## Conflict of Interest

The authors declare no conflict of interest.

## Author Contributions

L.C. and Y.L. contributed equally to this work. L.C. and Y.L. performed all experiments, analyzed the data, and drafted the manuscript. Y.Y., M.C., H.L., M.H., G.Y., J.G., H.W., W.S., and H.J. helped in project design, cellular assays, primary neuronal culture, immunostaining, and mice genotyping. Z.S. helped in RNA‐seq data and RIP‐seq data analysis. H.W. conceptualized the study, performed analyses, edited the manuscript with inputs from all authors. All authors have read and approved the final manuscript.

## Supporting information



Supporting Information

Supplemental Table 2

Supplemental Table 3

## Data Availability

The data that support the findings of this study are available from the corresponding author upon reasonable request.
