# Supplementary material for: EFTUD2 Regulates Cortical Morphogenesis via Modulation of Caspase‐3 and Aifm1 Splicing Pathways
Source: Adv Sci (Weinh). 2025 May 31;12(32):e04200. doi: 10.1002/advs.202504200 (PMC12407278; doi:10.1002/advs.202504200)
Supplement: Supplementary file 1 — Supporting Information [file ADVS-12-e04200-s003.docx]

**Supporting Information for**

**EFTUD2 Regulates Cortical Morphogenesis *via* Modulation of *Caspase-3* and *Aifm1* Splicing Pathways**

Liping Chen^1,#^, Ying Li^1,#^, Yan Yu^1^, Mingze Cai^1^, Hao Li^1^, Minghe Huang^1^, Guochao Yang^1^, Jiageng Guo^1^, Huailin Wang^1^, Zhihong Song^1^, Wei Shen^1^, Huihui Jiang^1^, Haitao Wu^1,2,3,^*

^1^Department of Neurobiology, Beijing Institute of Basic Medical Sciences, 100850 Beijing, China

^2^Key Laboratory of Neuroregeneration, Co-innovation Center of Neuroregeneration, Nantong University, Nantong, 226019 Jiangsu Province, China

^3^Chinese Institute for Brain Research, 102206 Beijing, China

^#^L.C. and Y.L. contributed equally to this work

^*^To whom correspondence may be addressed. E-mail: wuht@bmi.ac.cn


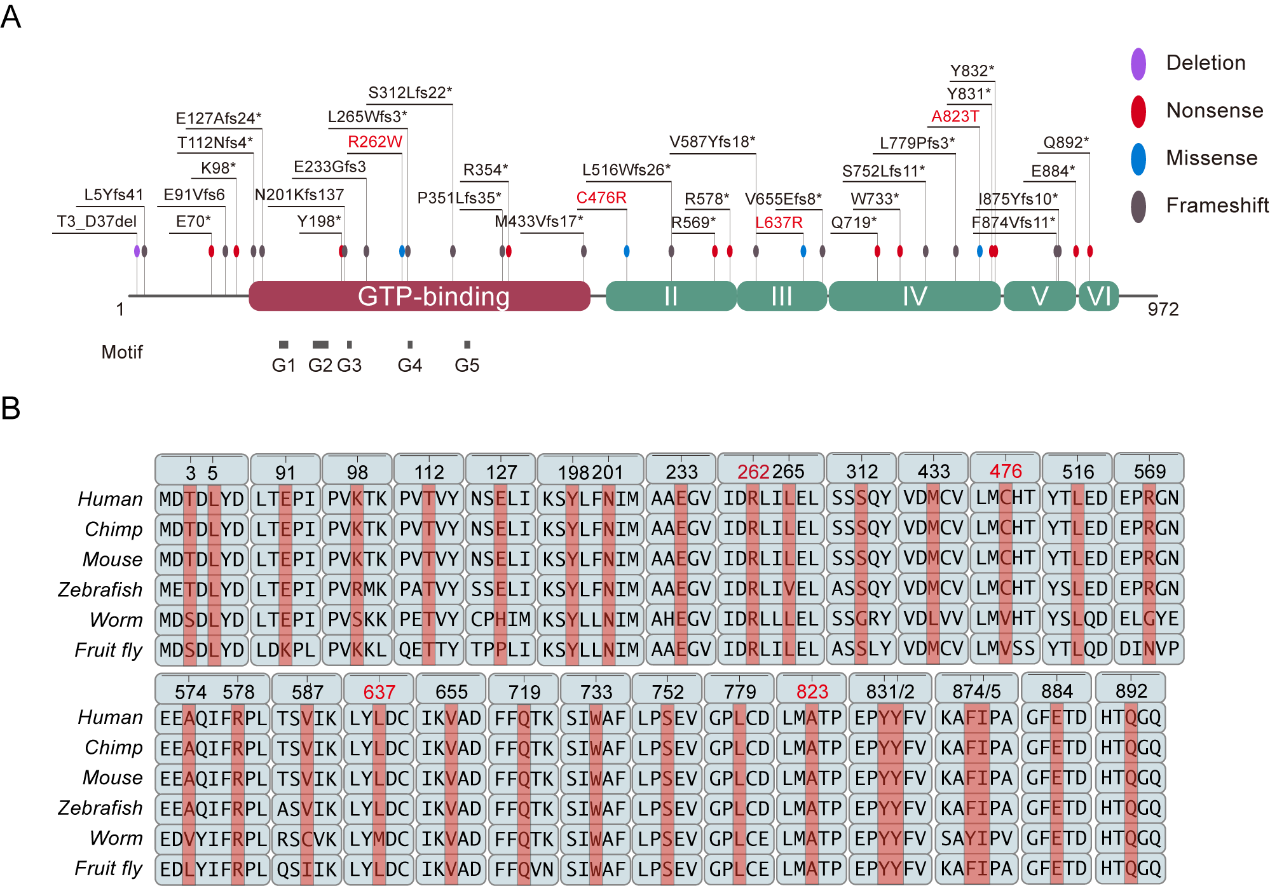


**Figure S1. *EFTUD2* variants identified in individuals with mandibulofacial dysostosis with microcephaly (MFDM).**

**A)** Domain architecture of human EFTUD2, highlighting critical functional regions, including the GTP-binding domain (red), II-VI domains (green). Missense variants (R262W, C476R, L637R, A823T) are denoted in blue; truncating variants (nonsense/frameshift) in red/gray and deletion variants in purple. **B)** Cross-species alignments of EFTUD2 protein sequences reveals that the missense variants (red) associated with MFDM occur at highly conserved residues. Species compared include humans (*Homo sapiens*, NP_003119.2), chimpanzees (*Pan troglodytes*, XP_001154155.1), mice (*Mus musculus*, NP_787030.2), zebrafish (*Danio rerio*, XP_009304586.2), worms (*C. elegans*, NP_001024053.2), and flies (*Drosophila melanogaster*, NP_001259660.1). The high degree of conservation underscores the functional importance of these residues across evolution.


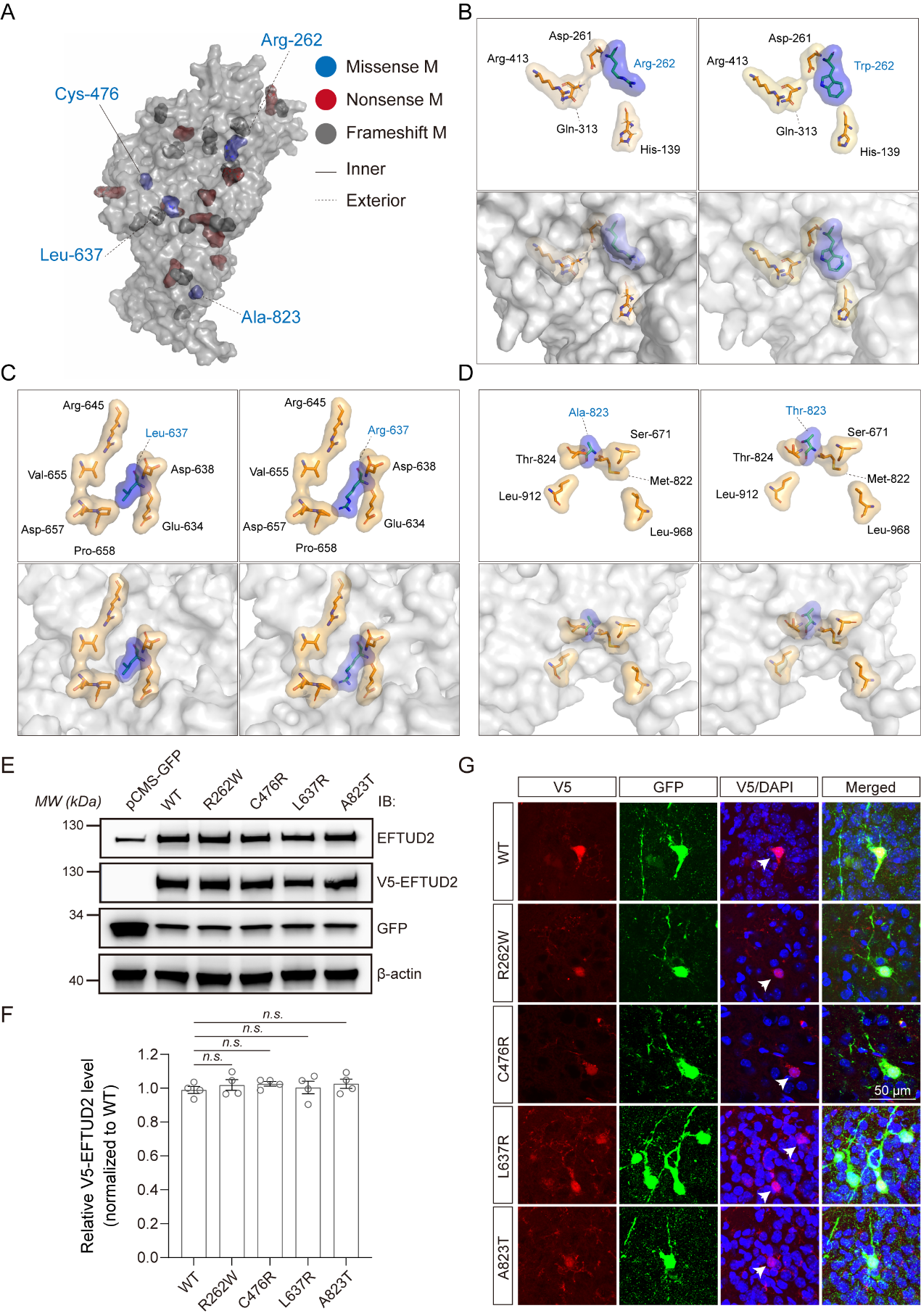


**Figure S2. Structure predictions and nuclear localization of pathogenic *EFTUD2* missense mutants.**

**A)** Predicted three-dimensional structure of human EFTUD2 (residues 114–957) generated via AlphaFold2 (AF-Q15029-F1), aligned to *Saccharomyces cerevisiae* elongation factor 2 (eEF2; PDB: 1N0U). Pathogenic missense variants localize to distinct structural regions: R262 (GTP-binding domain surface), L637 (HEAT repeat interface), A823 (β-propeller solvent-exposed loop), and C476 (hydrophobic core). **B-D)** Structural perturbations caused by the R262W (B), L637R (C), and A823T (D) mutations in EFTUD2. **E, F)** Representative immunoblot (E) and quantification (F) of V5-tagged EFTUD2 in HEK293T cells transfected with plasmids overexpressing wild-type (WT) *EFTUD2* or its mutants. Mutants showed no significant changes in protein expression levels (n = 4; one-way ANOVA and Dunnett’s multiple comparisons test). **G)** Co-localization assay of V5-tagged EFTUD2 and DAPI in GFP^+^ control neurons or neurons electroporated with WT or mutant human *EFTUD2* at E14.5. Analysis at P7 revealed preserved nuclear localization in all mutants. Scale bar: 50 μm.

All data represent means ± SEM. n.s., not significant.


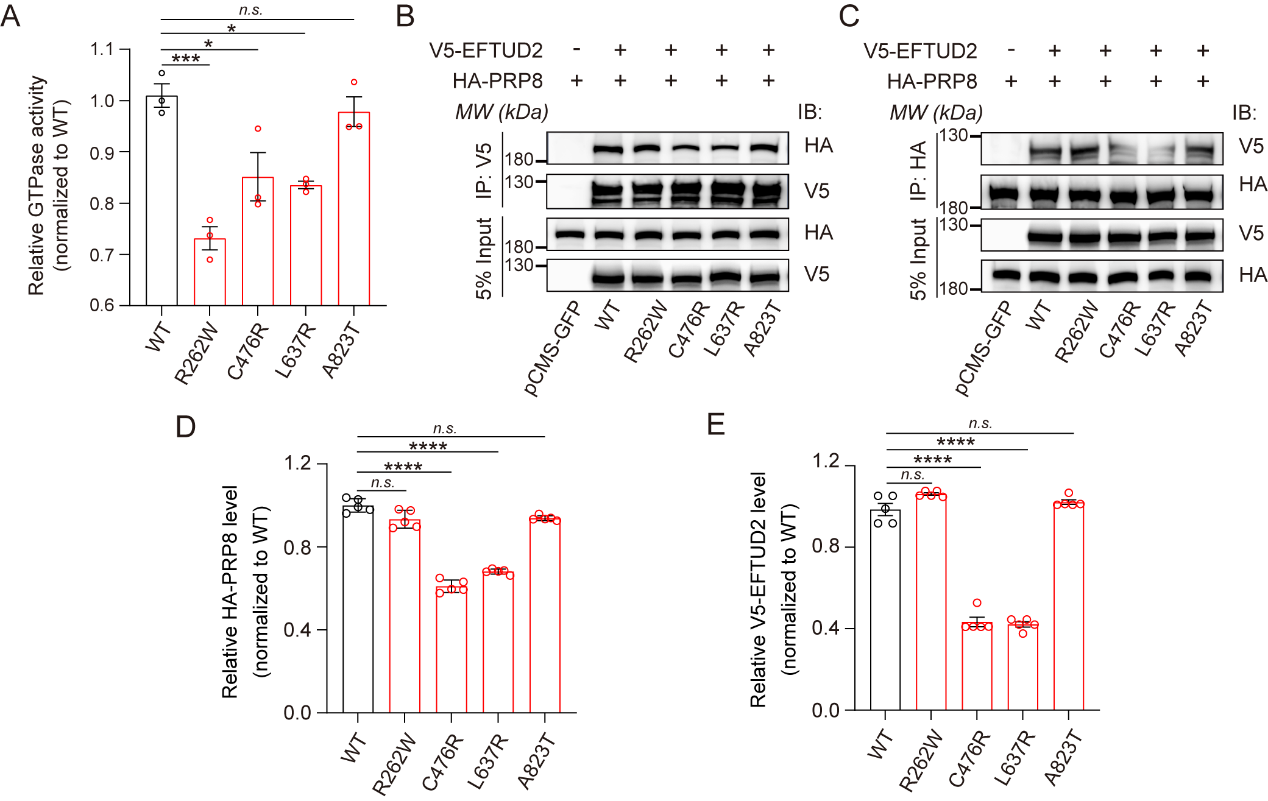


**Figure S3. Pathogenic *EFTUD2* missense mutants attenuate GTPase activity and PRP8 interaction.**

**A)** Comparative analysis of GTPase activity in immunoprecipitated pathogenic *EFTUD2* missense mutants relative to wild-type (WT) protein. HEK293T cells were transiently transfected for 48 hours with V5-tagged WT or mutant *EFTUD2* constructs (n = 3; one-way ANOVA with Dunnett’s post-*hoc* test). **B, C)** Co-immunoprecipitation (co-IP) assays performed in HEK293T cells co-transfected with V5-tagged *EFTUD2* (WT or mutants) and HA-tagged *PRP8*. Co-IP was conducted using anti-V5 (B) or anti-HA (C) antibodies. **D, E)** Quantification of co-IP complexes normalized to input levels (n = 5; one-way ANOVA with Dunnett’s post-*hoc* test).

All data represent means ± SEM. **P* < 0.05, ****P* < 0.001, *****P* < 0.0001, n.s., not significant.


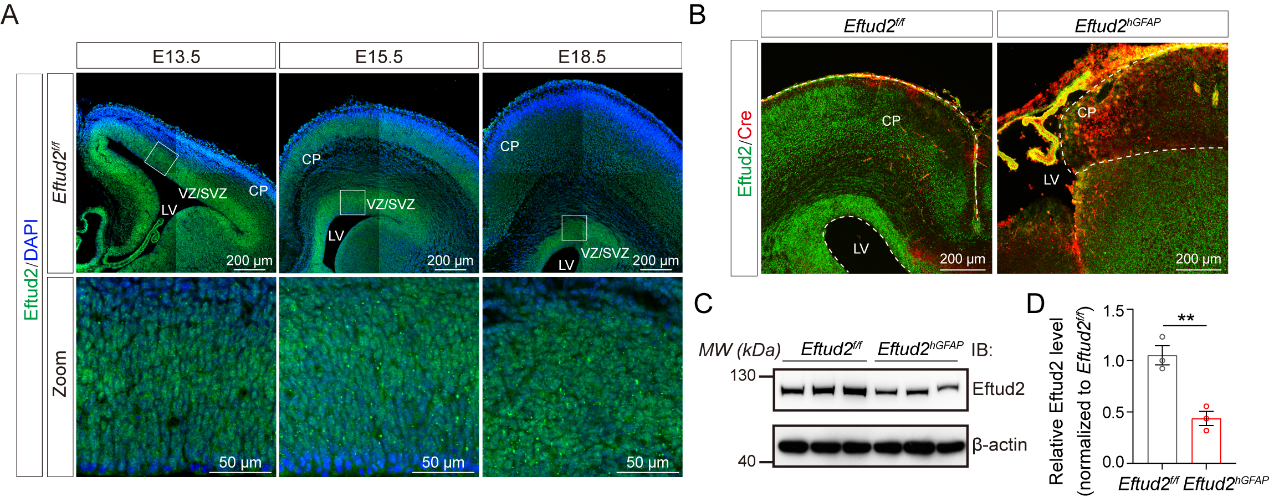


**Figure S4. Downregulated Eftud2 expression in the neocortex of *Eftud2^hGFAP^* cKO mice**

**A)** Representative immunofluorescence images of cerebral cortex at different embryonic stages showing Eftud2 expression in *Eftud2^f/f^* controls. Sections were co-stained with anti-Eftud2 antibodies. Scale bars: 200 μm or 50 μm. **B)** Representative immunofluorescence images of the cortical plate (CP) at postnatal day 0 (P0) showing reduced Eftud2 expression in *Eftud2^hGFAP^* cKO mice compared to *Eftud2^f/f^* controls. Sections were co-stained with anti-Cre (to validate recombination) and anti-Eftud2 antibodies. Scale bar: 200 μm. **C, D)** Immunoblot validation of Eftud2 protein levels in cortical lysates from *Eftud2^f/f^* controls and *Eftud2^hGFAP^* cKO mice. (B) Representative immunoblot and (C) quantitative analysis confirm a significant reduction in Eftud2 expression in cKO mice at P0 (n = 3; unpaired *t*-test).

All data represent means ± SEM. ***P* < 0.01. Abbreviations: CP, cortical plate; LV, lateral ventricle; VZ, ventricular zone; SVZ, subventricular zone.


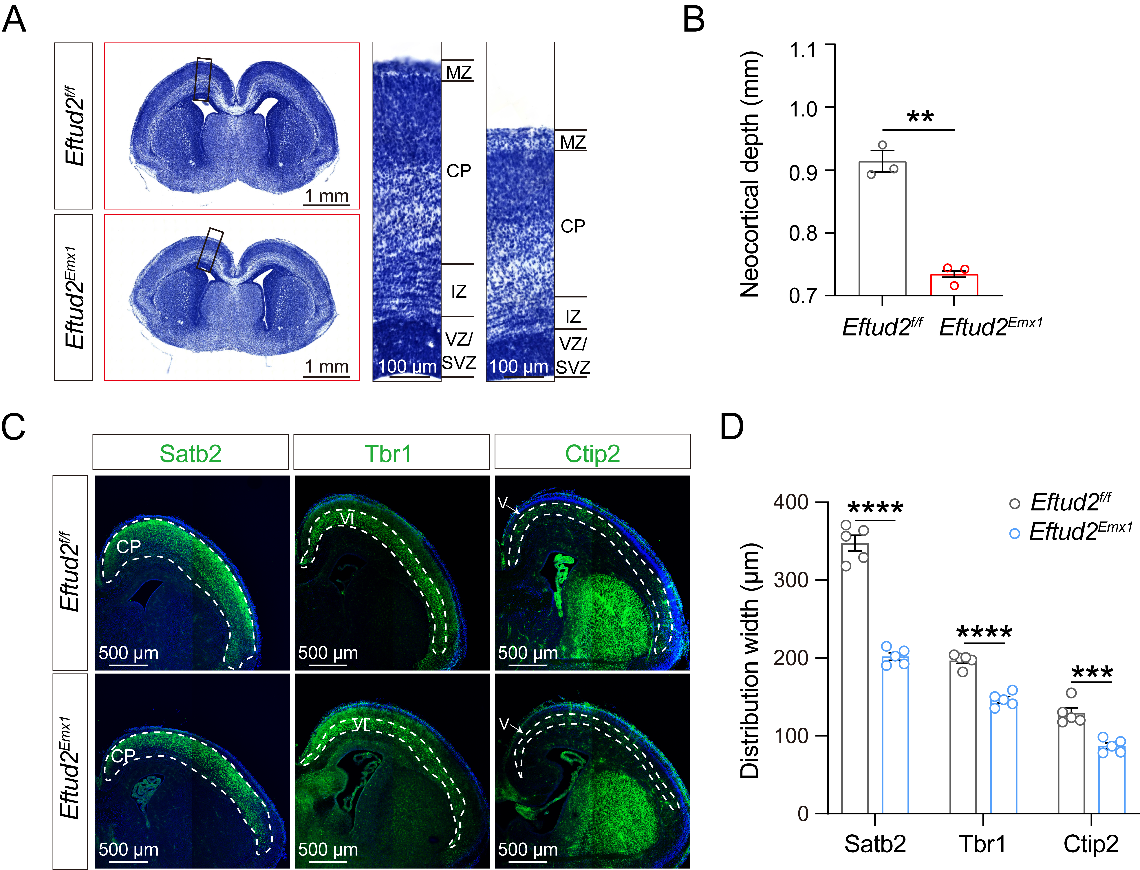


**Figure S5. Cortical structural abnormalities in *Eftud2^Emx1^* cKO mice**

**A)** Nissl-stained coronal brain sections at P0 demonstrate reduced neocortical thickness in the lateral cortices of *Eftud2^Emx1^* cKO mice compared to *Eftud2^f/f^* controls. Insets highlight magnified regions (scale bars: 1 mm for whole sections; 100 μm for insets). **B)** Quantification of neocortical depth confirms significant thinning in cKO mice (n = 3 mice; unpaired *t*-test). **C)** Immunofluorescence analysis of cortical layer markers at P0 reveals disrupted expression of deep-layer markers Tbr1 (layer VI) and Ctip2 (layer V), and pan-neocortical marker Satb2 in cKO cortices (scale bars: 500 μm). **D)** Quantification of neuronal density and laminar distribution width per unit area shows significant reductions in cKO mice (n = 5 mice; two-way ANOVA with Sidak’s multiple comparisons test).

All data represent means ± SEM. ***P* < 0.01, ****P* < 0.001, *****P* < 0.0001. Abbreviations: MZ, marginal zone; CP, cortical plate; IZ, intermediate zone; VZ, ventricular zone; SVZ, subventricular zone.


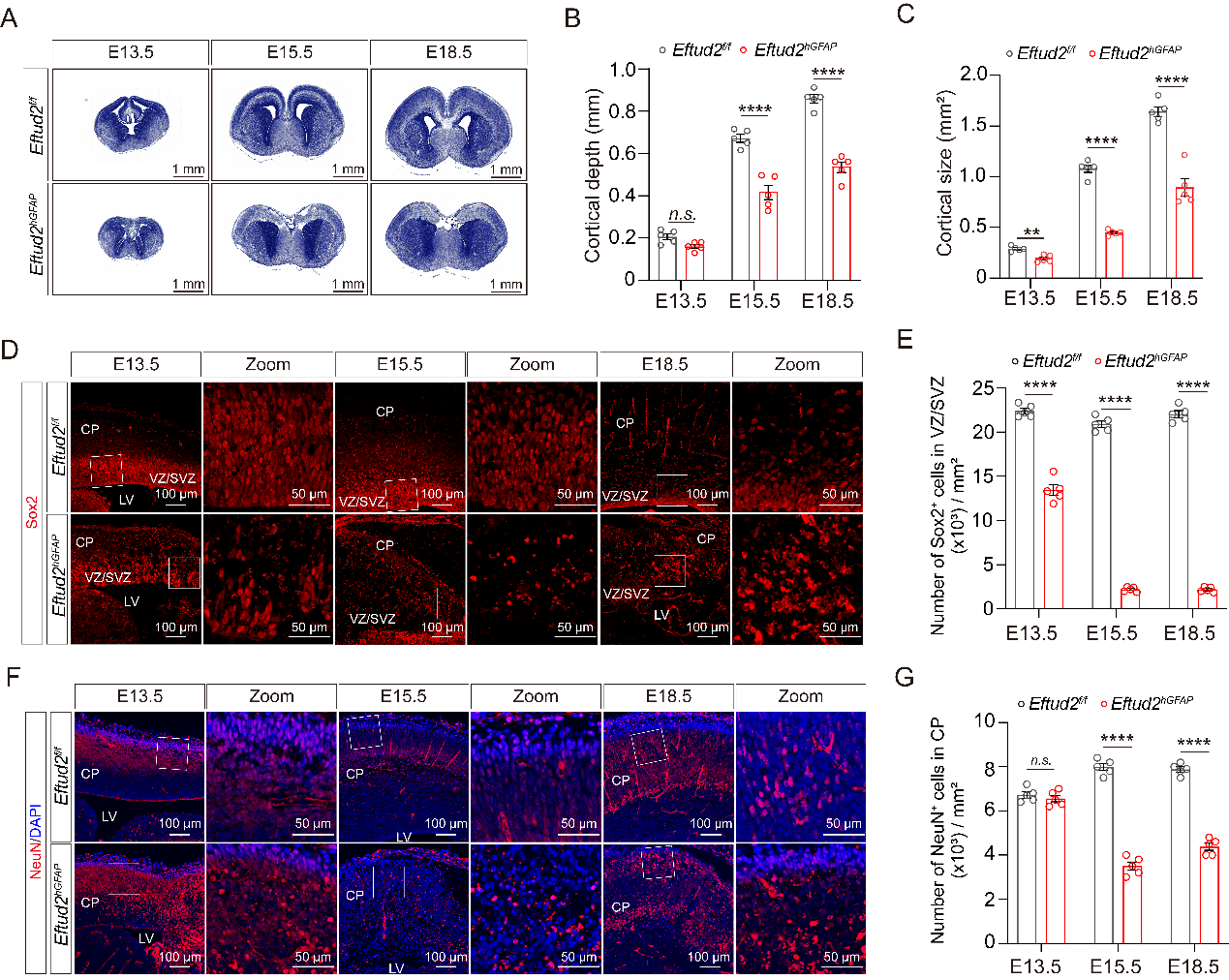


**Figure S6. *Eftud2* knockout disrupts NSC maintenance and neurogenesis in developing cerebral cortex in mice**

**A)** Nissl-stained coronal sections of embryonic cortices (E13.5, E15.5, E18.5) demonstrate progressive thinning of lateral cortical regions in *Eftud2^hGFAP^* cKO mice compared to *Eftud2^f/f^* controls. Scale bar: 1 mm. **B, C)** Quantification of (B) cortical depth and (C) total cortical area across developmental stages confirms significant reductions in cKO mice (n = 5 mice; two-way ANOVA with Sidak’s multiple comparisons test). **D)** Immunofluorescence analysis of NSCs using Sox2 reveals attenuated NSC populations in cKO cortices at E13.5, E15.5, and E18.5 (scale bars: 100 μm for overview; 50 μm for magnified regions). **E)** Quantification of Sox2^+^ NSCs per unit area shows robust depletion in cKO mice at all developmental stages (n = 5 mice; two-way ANOVA with Sidak’s multiple comparisons test). **F)** Immunofluorescence staining for mature neurons (NeuN) highlights reduced neuronal density in cKO cortices (scale bars: 100 μm and 50 μm). (G) Quantification of NeuN^+^ neurons confirms significant deficits in cKO mice compared to controls (n = 5 mice; two-way ANOVA with Sidak’s multiple comparisons test).

All data represent means ± SEM. ***P* < 0.01, *****P* < 0.0001, n.s., not significant. Abbreviations: CP, cortical plate; LV, lateral ventricle; VZ, ventricular zone; SVZ, subventricular zone.


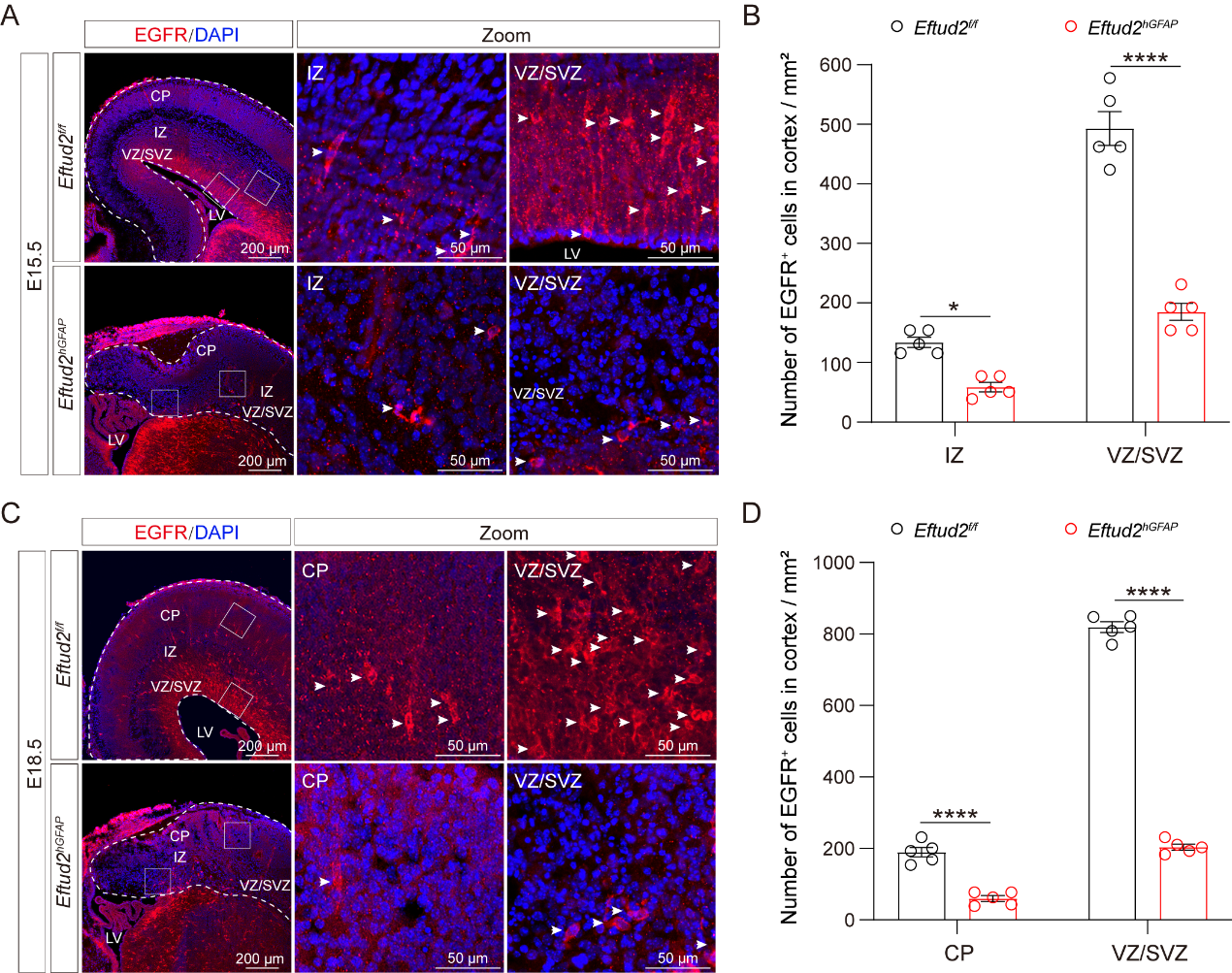


**Figure S7. *Eftud2* knockout induces the loss of glial cell progenitors in developing cerebral cortex of mice**

**A)** Immunofluorescence analysis of glial cell progenitors using EGFR reveals attenuated EGFR^+^ cells in cKO cortices at E15.5 (scale bars: 200 μm for overview; 50 μm for magnified regions). **B)** Quantification of EGFR^+^ glial cell progenitors per unit area shows robust depletion in cKO mice at E15.5 (n = 5 mice; two-way ANOVA with Sidak’s multiple comparisons test). **C)** Immunofluorescence analysis of glial cell progenitors using EGFR reveals attenuated EGFR^+^ cells in cKO cortices at E18.5 (scale bars: 200 μm for overview; 50 μm for magnified regions). **D)** Quantification of EGFR^+^ glial cell progenitors per unit area shows robust depletion in cKO mice at E18.5 (n = 5 mice; two-way ANOVA with Sidak’s multiple comparisons test).

All data represent means ± SEM. **P* < 0.05, *****P* < 0.0001. Abbreviations: CP, cortical plate; LV, lateral ventricle; IZ, intermediate zone; VZ, ventricular zone; SVZ, subventricular zone.


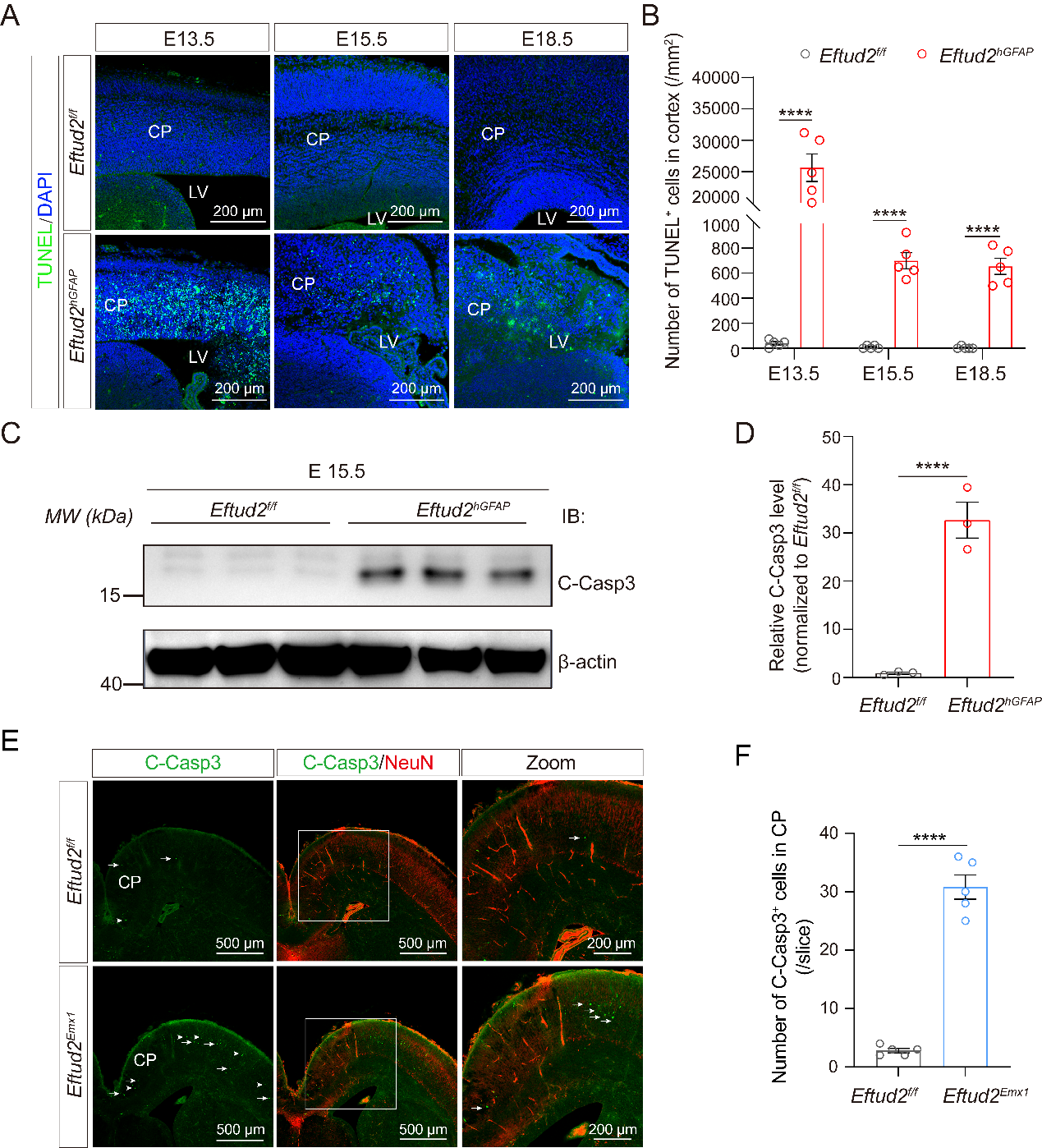


**Figure S8. *Eftud2* knockout induces cortical apoptosis in *Eftud2^hGFAP^* and *Eftud2^Emx1^* cKO mice**

**A)** TUNEL staining of embryonic cortices (E13.5, E15.5, E18.5) reveals elevated apoptotic cell density in *Eftud2^hGFAP^* cKO mice compared to *Eftud2^f/f^* controls. Scale bar: 200 μm. **B)** Quantification of TUNEL^+^ cells confirms a significant increase in apoptosis across all developmental stages in *Eftud2^hGFAP^* cKO cortices (n = 5 mice; two-way ANOVA with Sidak’s multiple comparisons test). **C, D)** Immunoblot analysis of C-Casp3 (C) and quantitative densitometry (D) demonstrate enhanced apoptotic signaling in *Eftud2^hGFAP^* cKO cortices at P0 (n = 3; unpaired *t*-test). **E)** Immunofluorescence staining for C-Casp3 in *Eftud2^Emx1^* cKO cortices at P0 shows increased apoptosis compared to controls (scale bars: 500 μm for whole sections; 200 μm for magnified regions). **F)** Quantification of C-Casp3^+^ cells validates significant apoptosis in *Eftud2^Emx1^* cKO mice (n = 5 mice; unpaired *t*-test).

All data represent means ± SEM. *****P* < 0.0001. Abbreviations: CP, cortical plate; LV, lateral ventricle.


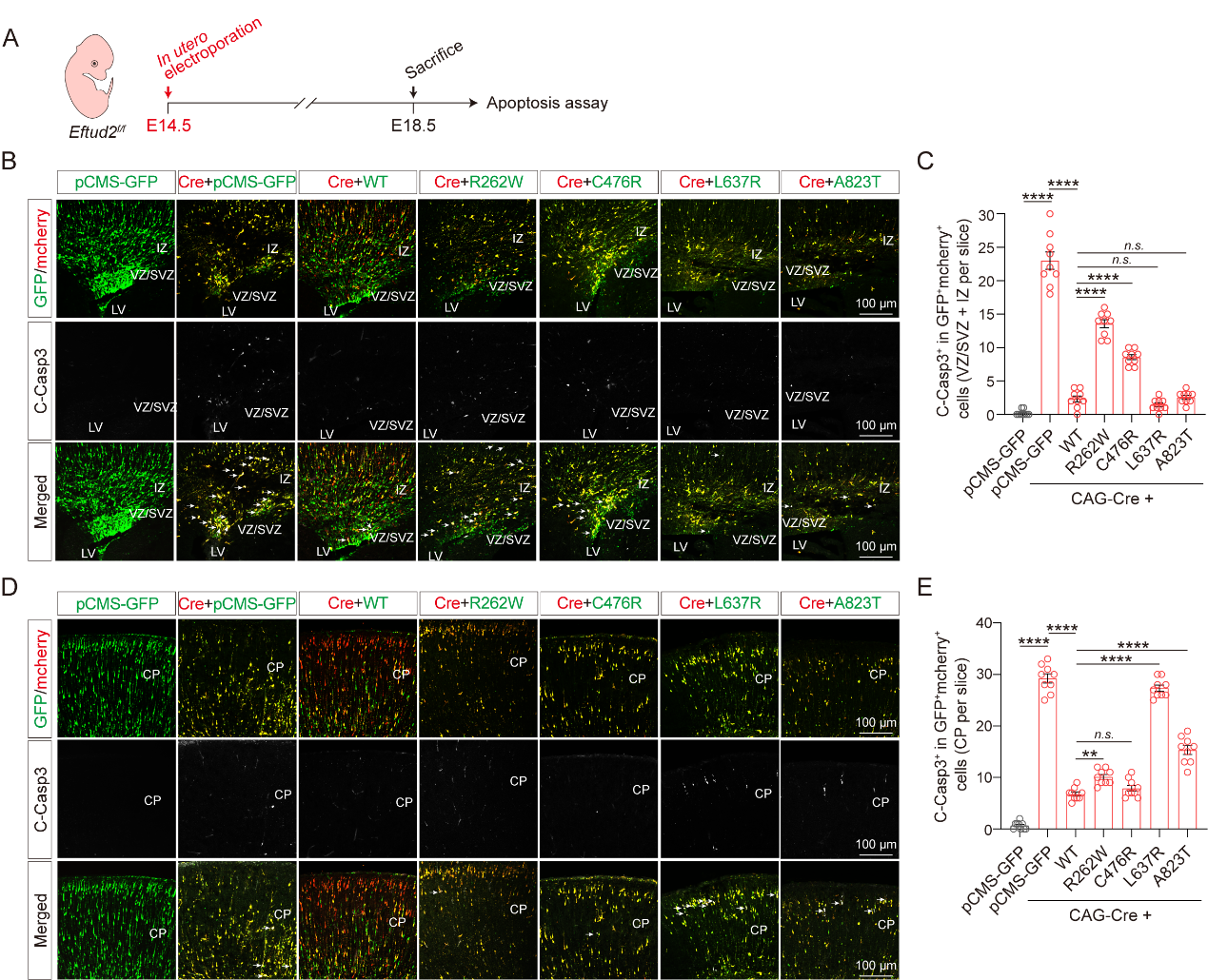


**Figure S9. Human *EFTUD2* pathogenic variants trigger apoptosis in cortical NSCs and neurons**

**A)** Schematic diagram of the experimental design and timeline for *in utero* electroporation. **B, D)** Representative immunofluorescence images of embryonic cortices showing co-localization of C-Casp3^+^ apoptotic cells with mCherry-Cre^+^ (*Eftud2* cKO) or GFP^+^ (control or human *EFTUD2*/*EFTUD2* variant-transfected) cells in the VZ/SVZ and IZ (B) and CP (D). Scale bar: 100 μm. **C, E)** Quantification of apoptotic cells (C-Casp3^+^) within mCherry^+^ (*Eftud2* cKO), GFP^+^ (transfected), and mCherry^+^GFP^+^ double-positive populations in the VZ/SVZ and IZ (C) and CP (E), demonstrating significantly elevated apoptosis in cortices expressing *EFTUD2* pathogenic variants (n = 9 mice; one-way ANOVA and Tukey multiple comparisons test).

All data represent means ± SEM. ***P* < 0.01, *****P* < 0.0001, n.s., not significant. Abbreviations: CP, cortical plate; LV, lateral ventricle; IZ, intermediate zone; VZ, ventricular zone; SVZ, subventricular zone.


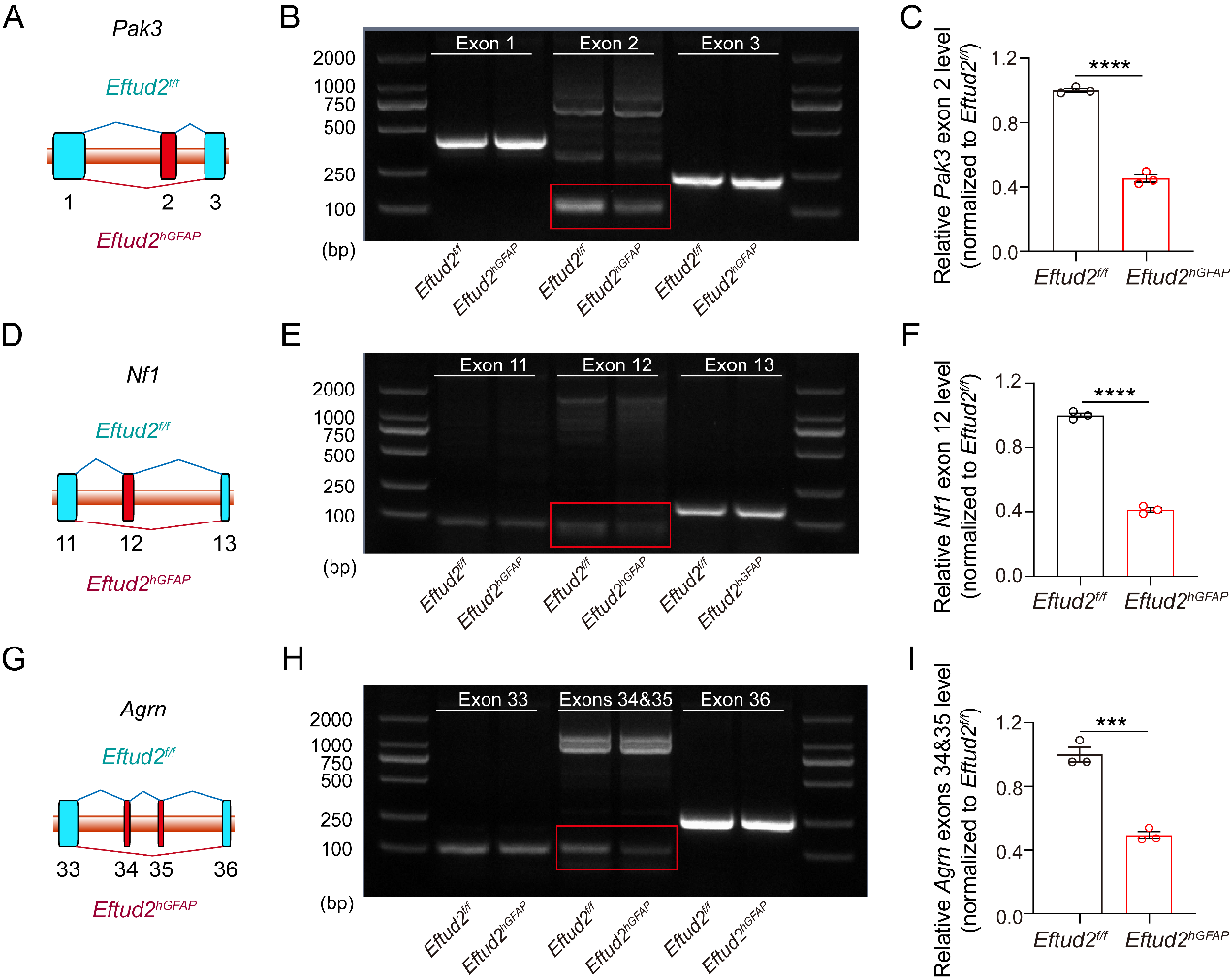


**Figure S10. Eftud2 regulates alternative splicing of apoptosis-related genes in the cortex of *Eftud2^hGFAP^* mice**

**A)** Alternative splicing of *Pak3* transcripts, showing exon 2 skipping in the cortex of *Eftud2^hGFAP^* cKO mice. **B, C)** RT-PCR analysis (B) and quantification (C) confirming increased exon 2 skipping in *Pak3* (n = 3; unpaired *t*-test). **D)** Alternative splicing of *Nf1* transcripts, demonstrating exon 12 skipping in *Eftud2^hGFAP^* cKO cortical tissue. **E, F)** RT-PCR validation (E) and quantitative analysis (F) of *Nf1* exon 12 skipping (n = 3; unpaired *t*-test). **G)** Alternative splicing of *Agrn* transcripts, revealing skipping of exons 34 and 35 in *Eftud2^hGFAP^* cKO mice. **H, I)** Representative RT-PCR data following electrophoresis (H) and quantification (I) confirming coordinated skipping of exons 34 and 35 in *Agrn* (n = 3; unpaired *t*-test)

All data represent mean ± SEM. ****P* < 0.001, *****P* < 0.0001.


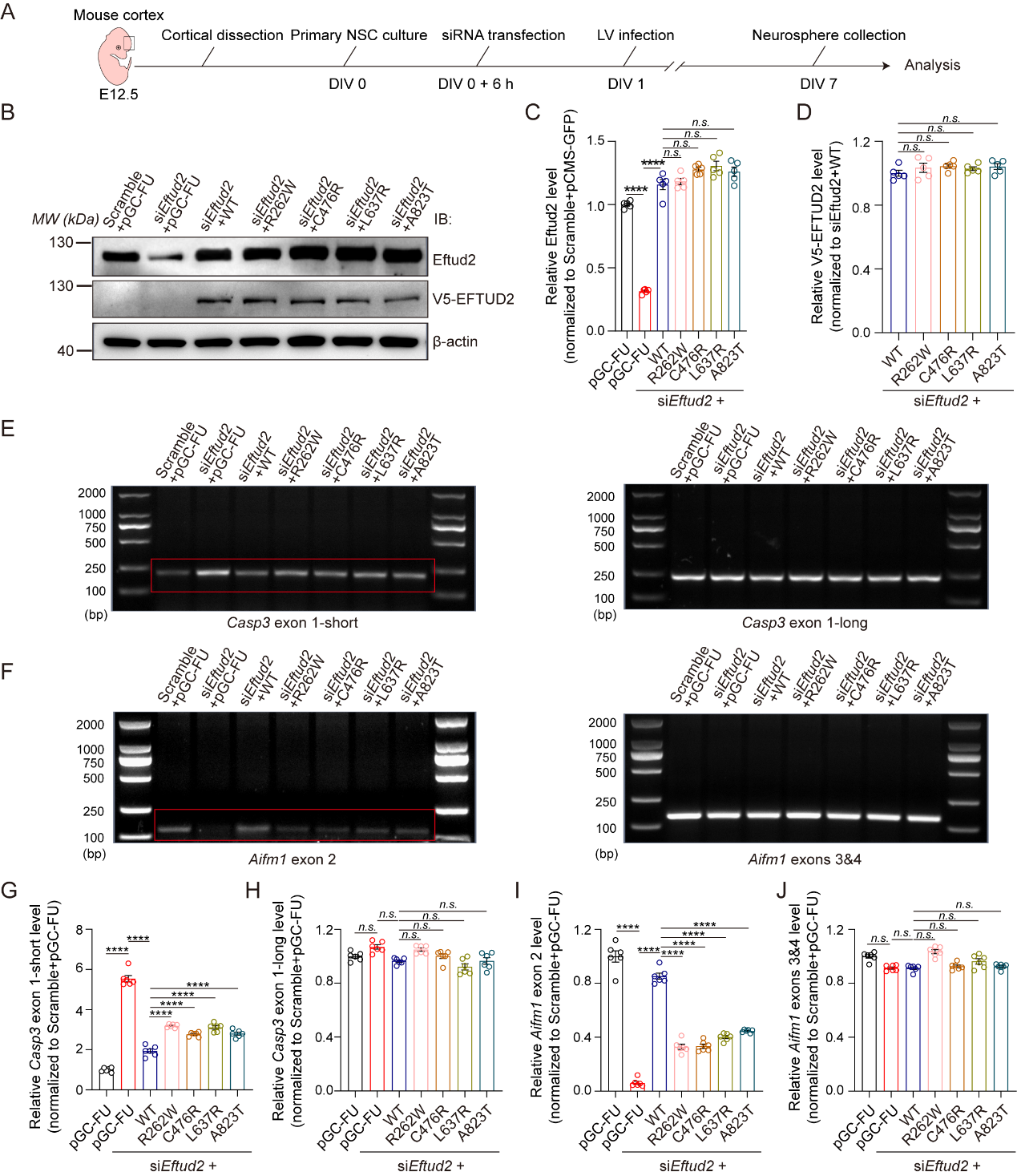


**Figure S11. Human *EFTUD2* pathogenic variants induce apoptosis in NSCs and alter the splicing of *Caspase3* and *Aifm1***

**A)** Schematic diagram of the experimental timeline and procedure for analyses in panels B-J. **B-D)** Immunoblot (B) and quantification of mouse Eftud2 (C) and V5-tagged human EFTUD2 (D) protein levels in NSCs 7 days after infection with Scramble RNA, si*Eftud2*, or si*Eftud2* co-expressed with WT *EFTUD2* or *EFTUD2* mutants (n = 5; one-way ANOVA and Tukey multiple comparisons test). **E, G, H)** RT-PCR validation (E) and quantification of *caspase3* short (G) or long (H) exon 1 in neurospheres at DIV 6 (n = 6, one-way ANOVA and Tukey multiple comparisons test). **F, I, J)** RT-PCR validation (F) and quantification of *Aifm1* exon 2 (I) and exons 3&4 (J) in neurospheres at DIV 6 (n = 6; one-way ANOVA and Tukey multiple comparisons test).

All data represent mean ± SEM. *****P* < 0.0001. n.s., not significant.


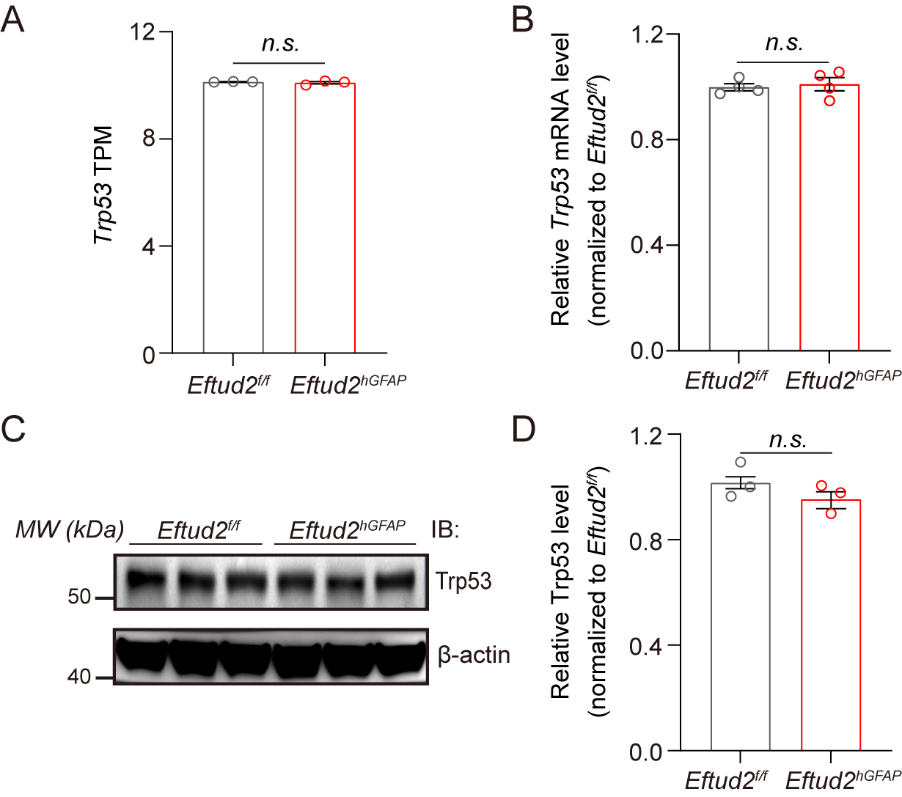


**Figure S12. Trp53 expression is unaltered in the cortex of *Eftud2^hGFAP^* cKO mice**

**A)** Transcripts Per Million (TPM) of *Trp53* transcript levels in the cortex of *Eftud2^f/f^* control and *Eftud2^hGFAP^* cKO mice at P0, showing no significant differences by RNA-sequencing (RNA-seq) analysis (n = 3; unpaired *t*-test). **B)** RT-qPCR validation of *Trp53* mRNA levels in cortical lysates from *Eftud2^f/f^* control and *Eftud2^hGFAP^* cKO mice (n = 4; unpaired *t*-test). **C, D)** Representative immunoblot (C) and quantitative analysis (D) of Trp53 protein levels in cortical tissues from *Eftud2^f/f^* control and *Eftud2^hGFAP^* cKO mice at P0, demonstrating comparable expression (n = 3; unpaired *t*-test).

All data represent means ± SEM. n.s., not significant.


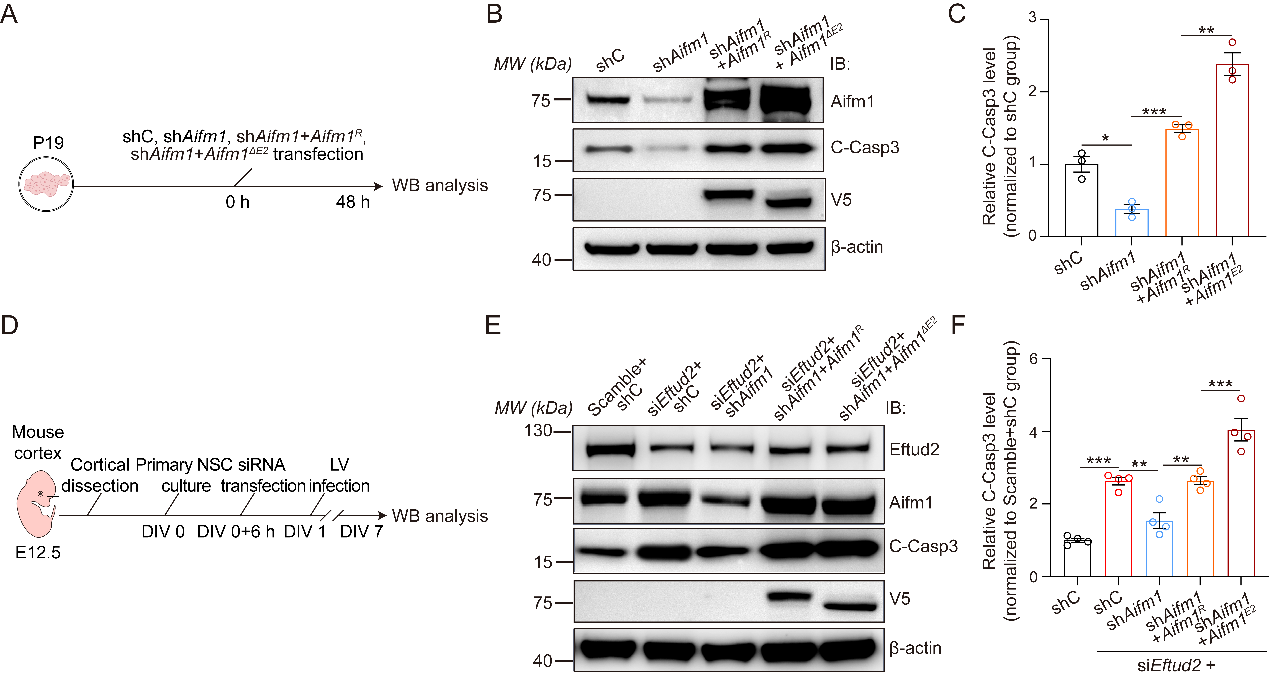


**Fig. S13. Exon 2 skipping in *Aifm1* enhances Caspase3 activation and apoptosis in cultured P19 cells and primary NSCs**

**A)** Experimental timeline and workflow for analyses in panels B-C. **B, C)** Western blot analysis (B) and quantification (C) of C-Casp3 levels in P19 cells transfected with FUGW-GFP plasmids. Exon 2 skipping in *Aifm1* significantly increased C-Casp3 levels (n = 3; one-way ANOVA with Tukey’s multiple comparisons test). **D)** Experimental timeline and workflow for panels E–F. **E, F)** Western blot analysis (E) and quantification (F) of C-Casp3 levels in cortical NSCs transduced with FUGW-GFP lentivirus (with or without siRNA). Exon 2 skipping in *Aifm1* significantly elevated C-Casp3 levels in lentivirus-treated NSCs (n = 4; one-way ANOVA with Tukey’s multiple comparisons test).

All data represent means ± SEM. **P* < 0.05, ***P* < 0.01, ****P* < 0.001.


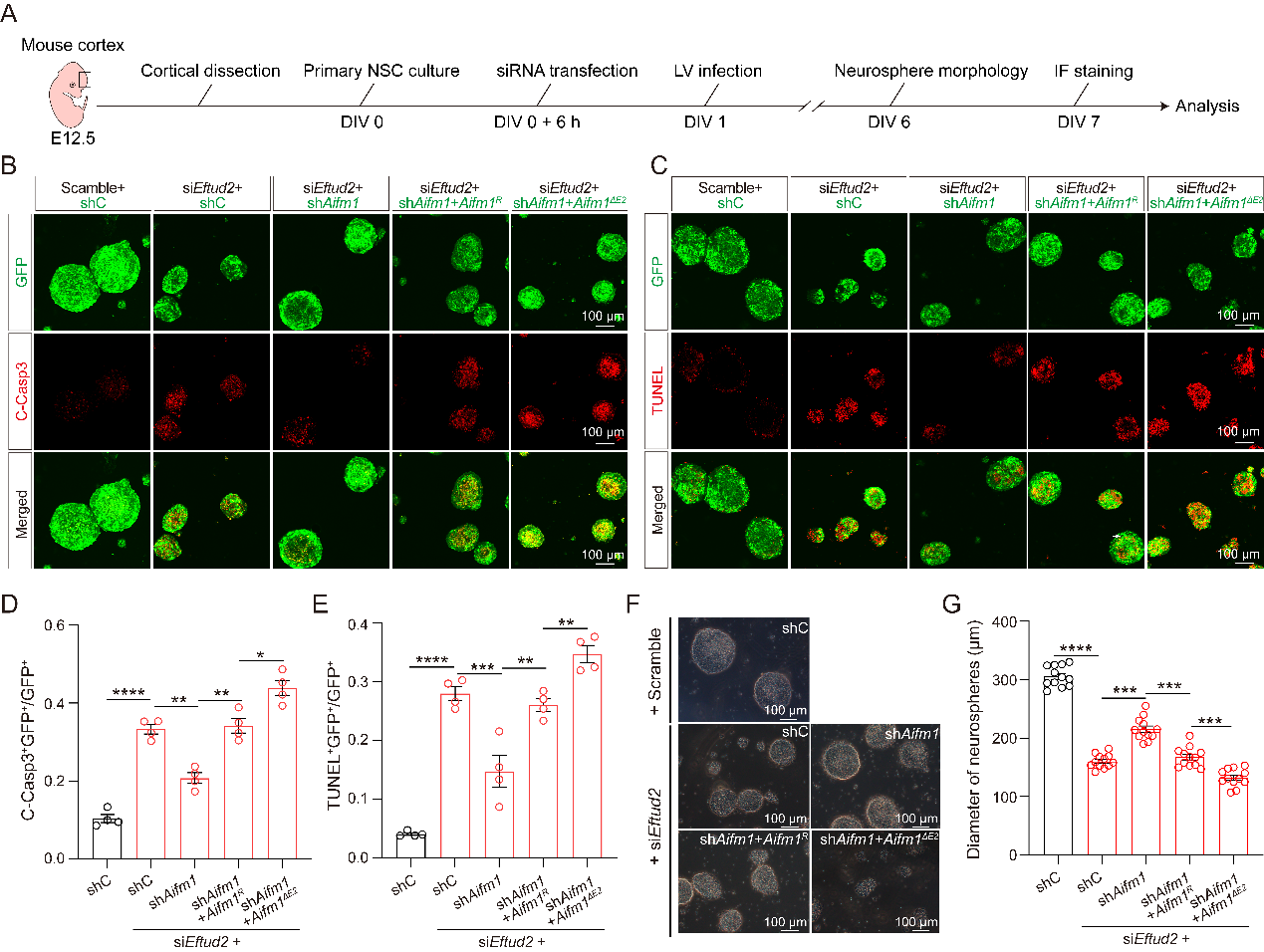


**Fig. S14 Exon 2 skipping in *Aifm1* promotes Caspase3 activation and augments apoptosis in cultured primary NSCs**

**A)** Schematic diagram of the experimental timeline and procedure for panels B-G. **B)** Immunofluorescent staining with anti-C-Casp3 overlaid on GFP^+^ NSC neurospheres at DIV 6. Scale bar: 100 μm. **C)** Immunofluorescent staining with TUNEL overlaid on GFP+ NSC neurospheres at DIV 6. Scale bar: 100 μm. **D, E)** Quantification of C-Casp3^+^GFP^+^/GFP^+^ (D), and TUNEL^+^GFP^+^/GFP^+^ (E) in NSC neurospheres at DIV 6 (n = 4; one-way ANOVA and Tukey multiple comparisons test). **F, G)** Distribution of the diameter of NSC neurospheres cultured for 6 days *in vitro* (DIV6) under differential interference contrast (DIC) microscopy (n = 12; one-way ANOVA and Tukey multiple comparisons test). Scale bar: 100 μm.

All data represent means ± SEM. **P* < 0.05, ***P* < 0.01, ****P* < 0.001, *****P* < 0.0001.

**Table S1. Primers used for genotyping and plasmid constructions**

| Primers | Sequence |
| --- | --- |
| Primer used for genotyping: *hGFAP*-Cre-F | GCCTGCATTACCGGTCGATGCAACGA |
| Primer used for genotyping: *hGFAP*-Cre-R | GTGGCAGATGGCGCGGCAACACCATT |
| Primer used for genotyping: *Emx1*-Cre-F | GCGGTCTGGCAGTAAAAACTATC |
| Primer used for genotyping: *Emx1*-Cre-R | GTGAAACAGCATTGCTGTCACTT |
| Primer used for genotyping: *Eftud2* loxP-F | GC AGGAAAGGTTAGCAGTC |
| Primer used for genotyping: *Eftud2* loxP-R | GTTCTCGTCGGTGGAATA |
| Primer used for genotyping: *Caspase3* mutant-1 | GCGAGTGAGAATGTGCATAAATTC |
| Primer used for genotyping: *Caspase3* mutant-2 | GGGAAACCAACAGTAGTCAGTCCT |
| Primer used for genotyping: *Caspase3* mutant-3 | TGCTAAAGCGCATGCTCCAGACTG |
| Primer used for pCMS-*EFTUD2*-WT-F | CGACTCACTATAGGCTAGCATGGATACCGACTTATATGATGAG |
| Primer used for pCMS-*EFTUD2*-WT-R | ACGCGTGAATTCTCGAGCTACGTAGAATCGAGACCGAG |
| Primer used for *EFTUD2* mutants: *EFTUD2* R262W-F | AGATTGACtGGCTGATCCTGGAGCTGAAGCTG |
| Primer used for *EFTUD2* mutants: *EFTUD2* R262W-R | GATCAGCCaGTCAATCTTGTTGATGCACACAGTG |
| Primer used for *EFTUD2* mutants: *EFTUD2* C476R-F | TGATGcGCCACACTACTAAGATGTACAGCACA |
| Primer used for *EFTUD2* mutants: *EFTUD2* C476R-R | AGTAGTGTGGCgCATCAGGGGGCCATCAGGGT |
| Primer used for *EFTUD2* mutants: *EFTUD2* L637R-F | TACCcGGACTGTGTGATGCATGATTTGCGGAA |
| Primer used for *EFTUD2* mutants: *EFTUD2* L637R -R | ATCACACAGTCCgGGTAGAGCTCCCCAGTGCCC |
| Primer used for *EFTUD2* mutants: *EFTUD2* A823T-F | CTGCCTTCCTCATGACTACTCCTCGTCTGATGG |
| Primer used for *EFTUD2* mutants: *EFTUD2* A823T-R | TCATGAGGAAGGCAGAGTAGACGACTCT |
| Sequence of H1-*Aifm1* shRNA targeting *Aifm1* mRNA | TTAATTAAAAAAAAGCTCTACTACTACGGCCAGATTCTCTTGAAATCTGGCCGTAGTAGTAGAGCGTGGTCTCATACAGAACTTATAAGATTCCCAAATCCAAAGACATTTCACGTTTATGGTGATTTCCCAGAACACATAGCGACATGCAAATATTGCAGGGCGCCACTCCCCTGTCCCTCACAGCCATCTTCCTGCCAGGGCGCACGCGCGCTGGGTGTTCCCGCCTAGTGACACTGGGCCCGCGATTCCTTGGAGCGGGTTGATGACGTCAGCGTTCTTAATTAA |
| Primer for sequencing H1-*Aifm1* shRNA | CTCGCTGCGCCCTTCGTCTGA |
| Sequence of *Aifm1* synonymous mutations on the shRNA targeting site: *Aifm1*-shRNA-F | TTAATTAAAAAAAAGCAACATGGTGAAACTTAATGCTCGAGCATTAAGTTTCACCATGTTGCCCGGGTGGTCTCATACAGAACTTATAAGATTCCCAAATCCAAAGACATTTCACGTTTATGGTGATTTCCCAGAACACATAGCGACATGCAAATATTGCAGGGCGCCACTCCCCTGTCCCTCACAGCCATCTTCCTGCCAGGGCGCACGCGCGCTGGGTGTTCCCGCCTAGTGACACTGGGCCCGCGATTCCTTGGAGCGGGTTGATGACGTCAGCGTTCTTAATTAA |
| Primer used for *Aifm1* exon 2 skipping variant (*Aifm1^ΔE2^*)-F | gcttccagcctacaaaactataaaagaagaccaaaaaag |
| Primer used for *Aifm1* exon 2 skipping variant (*Aifm1^ΔE2^*)-R | TTTTGTAGGCTGGAAGCCGGTTCCTCTGTTTC |
| Human *EFTUD2*-siRNA-1 | GATGGAGCCTTACTACTTT |
| Human *EFTUD2*-siRNA-2 | GAGCAGACATTACCTGTTA |
| Human *EFTUD2*-siRNA-3 | CAAGGCTCTTCTTGGTTCA |
| Mouse *Eftud2*-siRNA | CCTCCAACAGATGCTTATT |
| Primer used for pKH3-*PRP8*-F | AGGAGTTCGAACCCTCGAGGATATGGCCGGAGTGTTTCCTTATC |
| Primer used for pKH3-*PRP8*-R | AGAAAGCTGGGTTGCGGCCGCTCAGGCATACAGGTCCTCCC |
| Primers used for qRT-PCR: *Ctsd-F* | TGGGAATGGACATACCCCCT |
| Primers used for qRT-PCR: *Ctsd-R* | AGGGTCCAGCAACACTAAGC |
| Primers used for qRT-PCR: *Casp7-F* | GCCGTGGGAACGATGACC |
| Primers used for qRT-PCR: *Casp7-R* | GACGTCCATACCTGTCGCTT |
| Primers used for qRT-PCR: *Pidd1-F* | AGCGTGGCAGCTTCTATCAG |
| Primers used for qRT-PCR: *Pidd1-R* | GGGCGTAGGTCCAAGTTCAA |
| Primers used for qRT-PCR: *Xiap-F* | GGTCAGCCTCCTTAAACTTCGT |
| Primers used for qRT-PCR: *Xiap-R* | ACATTGCACGGTGTCTCCTT |
| Primers used for qRT-PCR: *Casp12-F* | TGATGAAGCCCACCTACCAC |
| Primers used for qRT-PCR: *Casp12-R* | GAAGGGGTGGCTCTCACAAT |
| Primers used for qRT-PCR: *Casp3-F* | GAGCTTGGAACGGTACGCTA |
| Primers used for qRT-PCR: *Casp3-R* | CCGTACCAGAGCGAGATGAC |
| Primers used for qRT-PCR: *Hrk-F* | CACTGTCTCTGCCATGGTGT |
| Primers used for qRT-PCR: *Hrk-R* | AAGGGAGTGATCATGCCAGC |
| Primers used for qRT-PCR: *Parp4-F* | ATCTCCGAGCAGGATGACTT |
| Primers used for qRT-PCR: *Parp4-R* | GTTGGCAATCTGCACATCGTT |
| Primers used for qRT-PCR: *Bak1-F* | AGACCCCATCCTGACCGTAA |
| Primers used for qRT-PCR: *Bak1-R* | GCTAAAGCGTCTTTGCCCTG |
| Primers used for qRT-PCR: *Ctsc-F* | AGAAGTTCCCGAAGCGACAT |
| Primers used for qRT-PCR: *Ctsc-R* | ATCATGGACCCACCCAGTCA |
| Primers used for qRT-PCR: *Dffb-F* | TGGCATGGCTATGTGAGTGA |
| Primers used for qRT-PCR: *Dffb-R* | CAGCAGCTTTTGCCTCAGTG |
| Primers used for qRT-PCR: *Bok-F* | TACGCAAGACCTTGGCTACC |
| Primers used for qRT-PCR: *Bok-R* | TACTGGAGGCCCCTTGGATT |
| Primers used for qRT-PCR: *p53-F* | GACCAAGAAGGGCCAGTCTAC |
| Primers used for qRT-PCR: *p53-R* | TGAGTGGAATCTGGGATTGTG |
| Primers used for qRT-PCR: *Actb-F* | GTGACGTTGACATCCGTAAAGA |
| Primers used for qRT-PCR: *Actb-R* | GCCGGACTCATCGTACTCC |
| Primers used for RT-PCR: *Casp3* exon 1 long*-F* | GGGGCTGAAACCACCAATC |
| Primers used for RT-PCR: *Casp3* exon 1 long*-R* | CATGGTCACTTTTCTTAGCGTAC |
| Primers used for RT-PCR: *Casp3* exon 1 short*-F* | GGGGCTGAAACCACCAATC |
| Primers used for RT-PCR: *Casp3* exon 1 short*-R* | CGTTCCAAGCTCCCCG |
| Primers used for RT-PCR: *Aifm1* exon 1 long*-F* | CTGAGTCTTACGCCTGC |
| Primers used for RT-PCR: *Aifm1* exon 1 long*-R* | CACCAGTTTCTGCTTGAAAG |
| Primers used for RT-PCR: *Aifm1* exon 2 long*-F* | TTGTGCAGTGTCTCC |
| Primers used for RT-PCR: *Aifm1* exon 2 long*-R* | ATAATAAATTCCTGCCCCAGT |
| Primers used for RT-PCR: *Aifm1* exon 3&4 short*-F* | GTGATGGGATTAGGACTGTCC |
| Primers used for RT-PCR: *Aifm1* exon 3&4 short*-R* | GCTGCAAAAGCAGCAGT |
| Primers used for RT-PCR: *Pak3* exon 1*-F* | AGGGTTTCACTTCCGCCTAGTT |
| Primers used for RT-PCR: *Pak3* exon 1*-R* | GTTTCAACCTCTTTGAGGCAGGAG |
| Primers used for RT-PCR: *Pak3* exon 2*-F* | CCCAGTACACCAGCCCATCT |
| Primers used for RT-PCR: *Pak3* exon 2*-R* | CACGTCTGAAGATGACTCAAAGCTG |
| Primers used for RT-PCR: *Pak3* exon 3*-F* | GAGCTGTGAAATCAGCTGCAACT |
| Primers used for RT-PCR: *Pak3* exon 3*-R* | TTTTATCCCCTCCTCCTGGGAAGAT |
| Primers used for RT-PCR: *Nf1* exon 11*-F* | ATCTGCCTGGCTCAGAATTCA |
| Primers used for RT-PCR: *Nf1* exon 11*-R* | ATTGGTGATGATGCGGTG |
| Primers used for RT-PCR: *Nf1* exon 12*-F* | GCACCGCATCATCACCAAT |
| Primers used for RT-PCR: *Nf1* exon 12*-R* | CCACCAATCCAACGCAGA |
| Primers used for RT-PCR: *Nf1* exon 13*-F* | TCTGCGTTGGATTGGTGG |
| Primers used for RT-PCR: *Nf1* exon 13*-R* | TGGTGCCATTCGTATTGCTG |
| Primers used for RT-PCR: *Agrn* exon 33*-F* | GGATAGTTGAGAAGTCAGTGGGGG |
| Primers used for RT-PCR: *Agrn* exon 33*-R* | CTCTCAGTCACAGCATTGAGGT |
| Primers used for RT-PCR: *Agrn* exons 34&35*-F* | ACCTCAATGCTGTGACTGAGAG |
| Primers used for RT-PCR: *Agrn* exons 34&35*-R* | GCTCTGCAGCGCTTTCTCA |
| Primers used for RT-PCR: *Agrn* exon 36*-F* | TGAGAAAGCGCTGCAGAGC |
| Primers used for RT-PCR: *Agrn* exon 36*-R* | CTGTGAGCCCTGACTCGAAG |
